# Cognitive impairment in psychiatric diseases: Biomarkers of diagnosis, treatment, and prevention

**DOI:** 10.3389/fncel.2022.1046692

**Published:** 2022-11-02

**Authors:** Yafen Wang, Weicheng Meng, Zhixin Liu, Qunxing An, Xingbin Hu

**Affiliations:** Department of Blood Transfusion, Xijing Hospital, Fourth Military Medical University, Xi’an, China

**Keywords:** cognitive impairment, psychiatric diseases, schizophrenia, bipolar disorder, autism spectrum disorder, major depressive disorder, biomarkers

## Abstract

Psychiatric diseases, such as schizophrenia, bipolar disorder, autism spectrum disorder, and major depressive disorder, place a huge health burden on society. Cognitive impairment is one of the core characteristics of psychiatric disorders and a vital determinant of social function and disease recurrence in patients. This review thus aims to explore the underlying molecular mechanisms of cognitive impairment in major psychiatric disorders and identify valuable biomarkers for diagnosis, treatment and prevention of patients.

## Introduction

Psychiatric diseases are the most enigmatic maladies in medicine, with various causes and limited effective treatment so far. Up to 20% of the population suffers from a common psychiatric disorder each year, especially adolescents aged 10–24 years (Jorm et al., 2017; GBD 2019 Diseases and Injuries Collaborators, 2020). Common psychiatric disorders include schizophrenia (SCZ), bipolar disorder (BD), autism spectrum disorder (ASD), and major depressive disorder (MDD), which together contribute significantly to the global burden of disability and disease (GBD 2019 Diseases and Injuries Collaborators, 2020). They are characterized by persistent, universal, and pathological patterns of abnormal moods, perceptions, behaviors, or higher levels of cognition (Sullivan and Geschwind, 2019; First et al., 2021). Of particular noteworthiness, cognitive impairment is a pivotal common characteristic of major psychiatric disorders, which can not only lead to dysfunction, disability and even death of patients, but also impose substantial psychological, economic, and medical burdens on families and society (Lai et al., 2014; Grande et al., 2016; Sharma et al., 2018; Vieta et al., 2018; Jauhar et al., 2022). There is, thus, a growing awareness of the importance of cognitive impairment.

The seven cognitive domains of cognitive impairment identified by MATRICS (Measurement and Treatment Research to Improve Cognition in Schizophrenia) Consensus Cognitive Battery (MCCB) typically include notable deficits in the speed of processing, working memory, attention/vigilance, verbal learning, visual learning, reasoning and problem-solving, and social cognition (Nuechterlein et al., 2008; Van Rheenen and Rossell, 2014; Lynham et al., 2022). An epidemiologic study of first-admission psychosis revealed that the prevalence of cognitive impairment in schizophrenia, bipolar disorder, and major depressive disorder was as high as 45, 64, and 77%, respectively (Reichenberg et al., 2009). Currently available therapeutic approaches and researches for cognitive impairment in psychiatric diseases rely on cognitive-enhancing agents and neuroplasticity-based cognitive training primarily (Guercio et al., 2019; Haas et al., 2021; Veselinović and Neuner, 2022). However, there are no objective molecular markers available to early diagnose, guide therapy and evaluate prognosis in existing clinical practice. Therefore, the development of novel and effective objective molecular markers became an urgent need to address limitations in the current.

**FIGURE 1:**
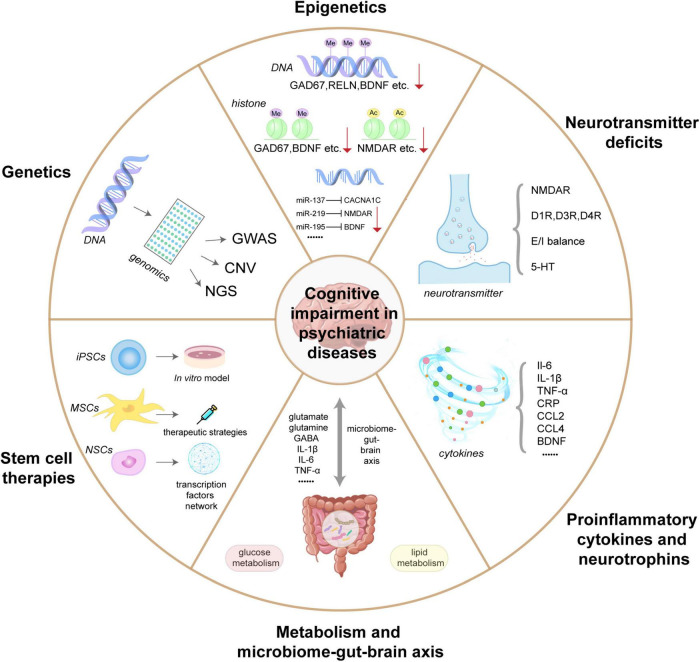
An overview of the molecular mechanisms underlying cognitive impairment in psychiatric diseases. Genetic studies of psychiatric disorders usually include genome-wide association studies (GWAS), rare copy number variation (CNV) studies, and Next generation sequencing (NGS) studies. Genetics relies on variation and allele frequency data to infer essential risk genes and population genetic analyses for cognitive function; Epigenetics of cognitive impairment in psychiatric disorders include DNA methylation (GAD67, RELN, BDNF, etc.), histone modifications (GAD67, BDNF, etc.), and various microRNAs (miR-137, miR-219, and miR-195), which typically inhibits the expression of their targets; Neurotransmitters deficits involved in cognitive impairment of psychiatric disorders include the glutamatergic system (NMDAR), the dopaminergic neurotransmitter system (D1R, D3R, and D4R), gamma-aminobutyric acid (GABA) system (E/I balance), and serotonin system (5-HT); Peripheral inflammatory factors and neurotrophic factors associated with cognitive impairment in psychiatric disorders include IL-6, IL-1β, TNF-α, CRP, BDNF, etc.; The metabolism and microbial gut-brain axis are critical bidirectional communication systems in cognitive impairment in psychiatric disorders; Stem cells (iPSCs, MSCs, and NSCs) provide an efficient *in vitro* modeling and therapeutic approach for the mechanism research of cognitive impairment in mental illness.

It is now known that manifold mechanisms contribute to understanding cognitive impairment in psychiatric diseases including genetics, epigenetics, neurotransmitter deficits, proinflammatory cytokines and neurotrophins, metabolism and microbiome-gut-brain axis, and stem cell therapies. An overview of this review is shown in Figure 1. The aim of the present review is, therefore, to systematically summarize and evaluate the pathogenesis of cognitive impairment in psychiatric diseases and to identify effective objective molecular markers for diagnosis and potential targets for future therapeutic approaches.

## Genetics

As psychiatric genomics continues to thrive, so do the unprecedented insights of cognitive impairment into psychiatric diseases. Genome-wide association studies (GWAS) has been used extensively in recent years to identify novel genetic loci associated with cognitive phenotype of multiple psychiatric conditions, especially in patients with SCZ (Lencz et al., 2014; Ohi et al., 2015; Smeland et al., 2017; Greenwood et al., 2019). It can identify sequence variation in the human genome and screen for single nucleotide polymorphisms (SNPs) associated with human disease phenotypes, thereby mining genomic regions associated with trait variation (Rees and Owen, 2020). Lencz et al. (2014) identified nominally significant cognitive associations for four SNPs that have previously been robustly associated with SCZ susceptibility and discovered polygenic risk scores for SCZ were associated with lower general cognitive ability. One year later, Ohi et al. revealed that 191 SNPs were related to the cognitive phenotype of SCZ, of which 115 SNPs (60.2%) have genes located within 10 kb of the SNPs. These genetic variants are moderately related to cognitive trait impairment and are primarily involved in the *N*-methyl-D-aspartate-glutamate (NMDA) network (Ohi et al., 2015). In recent years, Greenwood et al. used a conditional false discovery rate statistical approach, taking advantage of several large GWAS on SCZ and cognitive traits in more than 250,000 participants, 21 genomic regions were found to be shared between SCZ and cognitive traits (Smeland et al., 2017). Another GWAS on 11 SCZ-related neurophysiological and neurocognitive endophenotypes reported that a total of seven regions (3p21, 5p15, 5p12, etc.) exceeding conventional genome-wide significance thresholds were associated with various endophenotype defects, and genes such as neuregulin 3 (NRG3) and hyperpolarization activated cyclic nucleotide gated potassium channel 1 (HCN1) within these regions are primarily implicated in glutamate and axonal dysfunctions (Greenwood et al., 2019). In addition to SCZ, a GWAS of MDD and cognitive function found that there were 48, 116, and 32 SNPs significantly associated with executive function domains, processing speed, and global cognition, respectively. Some of these SNPs are located in genes expressed in brain with important roles in neuronal development (REST), oligodendrocyte maturation (TNFRSF21) and myelination (ARFGEF1) (Thalamuthu et al., 2022). A large GWAS combining data on SCZ (*n* = 82,315), BD (*n* = 51,710), and general intelligence (*n* = 269,867) identified 75 distinct genomic loci associated with SCZ and intelligence, and 12 loci associated with BD and intelligence. Most SCZ risk alleles (61 of 75, 81%) were associated with poor cognitive performance, whereas most BD risk alleles (9 of 12, 75%) were associated with better cognitive performance, revealing a broad genetic overlap between SCZ, BD, and intelligence, albeit in different ways (Smeland et al., 2020).

According to the above mentioned studies, GWAS is being increasingly used in the study of cognitive impairment in mental disorders due to its advantages of effective identification of genetic variation and public usability of data. These massive amount of genomics data provide novel information at an unprecedented scale, but how to use genetic data to develop neurobiological insights and new therapeutic targets is a question that needs to be considered. Candidate genes are typically identified based on their location (i.e., their proximity to loci associated with cognitive impairment in psychiatric disorders) or by integrating findings of GWAS with functional information and gene expression data (Mould et al., 2021). Shortlisting such genes could provide potential drug targets for improving cognitive impairment in patients with psychiatric disorders. For example, the genes involved in the GWAS study of SCZ are targets of drugs with approved indications, such as voltage-gated calcium channels (e.g., CACNA1C and CACNB2) (Lencz and Malhotra, 2015; Hidalgo et al., 2021). However, due to the complexity of the brain, the polygenic nature of cognitive impairment, and the uncertainty of pathogenic mechanisms, no single therapeutic target or strategy is likely to be effective in all or even most patients. Second, the functions of the individual genes involved in GWAS can be explored in animal models, but it is difficult to see how many human risk alleles can be modeled with current or emerging technologies (Lencz and Malhotra, 2015; Rees and Owen, 2020). As a result, the genetic mechanism of cognitive impairment in mental illness remains challenges.

In addition to GWAS, rare copy number variant (CNV) is another significant genomic study that identifies deletions or duplications of DNA base pairs associated with traits or diseases (Rees and Owen, 2020). Large (>100 kb), rare (<1% in the population) CNVs have been shown to confer risk of cognitive impairment in SCZ and ASD (Stefansson et al., 2014; Rees and Kirov, 2021). A CNV analysis of SCZ identified 34 *de Novo* CNVs, and gene-set enrichment analysis suggested that these CNVs may contribute to defects in *N*-methyl-D-aspartate receptor (NMDAR) postsynaptic signaling and neuronal activity-regulated cytoskeleton-associated protein (known to be important in synaptic plasticity and cognition) (Kirov et al., 2012). CNV carriers predisposed to SCZ and/or autism have cognitive abnormalities similar to those encountered in SCZ, so these neuropsychiatric CNVs can be used as tools to study the cognitive abnormalities that characterize the disorder (Stefansson et al., 2014). Growing evidence in recent years also clearly implicates rare CNVs hitting genes in SCZ-enriched gene sets were associated with severe cognitive impairment (Kendall et al., 2019a; Hubbard et al., 2021). High-risk CNV is not only related to SCZ and other neurodevelopmental disorders, but is also a factor contributing to impairment in cognitive domains such as memory or perceptual reasoning, and acts as an intermediate risk biomarker of disease (Thygesen et al., 2021). Similarly, autistic probands with multiple pathogenic CNVs and a strong family history presented more severe clinical features and impaired cognition (Sanders et al., 2015; Pizzo et al., 2019). However, from the current research, it appears that the contribution of large CNVs to BD and MDD is smaller than that to SCZ and ASD. Large structural variations often seem to predispose to persistent, widespread brain dysfunction, including impacts on the cognitive and personality development (Green et al., 2016; Kendall et al., 2019b).

Next-generation sequencing (NGS) studies can detect other rare variants, such as rare single nucleotide variants and small insertions/deletions. NGS often have focused on sequencing protein-coding regions of the genome, known as exome sequencing, to identify specific genes or genomes rich in rare variants associated with psychiatric disorders (Rees and Owen, 2020; Satterstrom et al., 2020). Recently, the advent of third-generation sequencing (TGS) technology has revolutionized our ability to sequence genomes and transcriptomes, and throw more lights on the complicate gene structure analysis (Sedlazeck et al., 2018). Compared to NGS technologies, TGS can generate longer sequencing reads, enabling the direct detection of DNA and RNA modifications at single-nucleotide resolution and in a single-molecule manner (van Dijk et al., 2018). It holds great potential for many fields, such as identification of variant-disease associations and methylation, and further promotion of disease diagnosis (Liu et al., 2020; Smith et al., 2020). The polygenic nature of psychiatric disorders challenges advances from genetics to biology. Still, by combining different genomics and functional, pharmaceutical, and clinical trial approaches, we will better understand the mechanism of cognitive impairment in mental disorders, explore better treatment methods, and judge the prognosis of patients.

## Epigenetics

Unlike genetics, epigenetics refers to heritable phenotypic changes that not alter the DNA sequence, including DNA methylation, histone modifications, and various microRNAs (Yao et al., 2016). Epigenetic regulation of cognitive impairment in mental illness has also attracted increasing attention. The PsychENCODE project is first the consortium to compare neuronal and non-neuronal transcriptomes and epigenomes from post-mortem brains of donors with psychiatric phenotypes, which assesses population epigenetic variation in the human brain and functionally characterizes variation associated with mental illness (Akbarian et al., 2015).

DNA methylation is one of the most studied epigenetic modifications in cognitive impairment, mainly refers to the methylation of CpG dinucleotides on cytosine dinucleotide 5-carbon atoms, which is usually associated with gene silencing (Grayson and Guidotti, 2013; Jaffe et al., 2016; Vogel Ciernia and LaSalle, 2016). Methylation studies of cognitive impairment in SCZ have focused on genes involved in the disease. Previous studies have reported that hypermethylation of the promoters of candidate genes expressed in cognitively related gamma-aminobutyric acid (GABA)-ergic neurons in SCZ is associated with transcriptional downregulation of corresponding mRNA, including glutamate decarboxylase 67 (GAD67/GAD1) and reelin (RELN) (Chen et al., 2002; Dong et al., 2005). Other common methylation candidate genes, include Catechol-O-methyltransferase (COMT), brain-derived neurotrophic factor (BDNF), dopamine receptor, and serotonin receptor (Viola and Fries, 2019). The COMT gene encodes Catechol-*O*-methyltransferase, which affects prefrontal dopamine levels and cognitive symptoms in SCZ (Ehlis et al., 2007). It has been suggested that the methylation of BDNF reduces its expression in SCZ patients, and this change affects the protein level of synaptic density and neuronal cell size and thus affects patients’ cognition, but there is some controversy (Ehlis et al., 2007; Boulle et al., 2012; Keller et al., 2014). Methylation of the promoter of dopamine receptor genes (DRD2, DRD3, and DRD4) also alters dopamine expression and causes cognitive deficits associated with SCZ (Cheng et al., 2014; Dai et al., 2014; Chen et al., 2020). Hypermethylation of genes (5HTR1A and HTR2A) that regulates serotonin signaling and serotonin transporter, which are involved in cognition and memory, has been found to be associated with SCZ (Carrard et al., 2011; Abdolmaleky et al., 2014; Cheah et al., 2017). In BD, methylation studies of candidate genes associated with cognitive impairment are similar to those in SCZ (Fries et al., 2016). Evidence for aberrant DNA methylation in ASD can be seen at multiple levels, ranging from gene mutations in epigenetic mechanisms to site-specific and genome-wide changes in DNA methylation (Vogel Ciernia and LaSalle, 2016). Targeted gene-specific DNA methylation changes in ASD included hypermethylation of MECP2, UBE3A, OXTR, SNRPN, MAGEL2, RELN, and GAD1. In contrast, hypomethylation of target candidate genes such as RORA, ERMN, USP24, METTL21C, PDE10A, STX16, and DBT was also found in peripheral blood DNA of ASD subjects (Tremblay and Jiang, 2019). Of these, RELN and GAD1 have been clearly associated with cognitive impairment (Labouesse et al., 2015; Lintas et al., 2016).

Histone modifications including methylation and acetylation of histones have recently been implicated in cognitive impairment in various psychiatric disorders (Peter and Akbarian, 2011; Jakovcevski and Akbarian, 2012; Girdhar et al., 2018). SCZ patients exhibited defective expression of histone H3K4-trimethylation (H3K4me3) and GABAergic promoter subsets of genes (GAD1, GAD2, NPY, and SST) in the prefrontal cortex, all of which are strongly associated with cognitive impairment (Huang et al., 2007; Gupta et al., 2010). Similarly, mature prefrontal neurons in mice are critically dependent on the maintenance of H3K4-specific methyltransferase-regulated H3K4 methylation which play essential roles in cognition and emotion (Jakovcevski et al., 2015; Javidfar et al., 2017). Thus, GAD1-bound H3K4me3 can be viewed as a molecular link that connects three key factors in SCZ neurobiology–GABA neuronal dysfunction, developmental mechanisms, and molecular responses following antipsychotics (Mitchell et al., 2015). Postmortem brain studies have confirmed that the expression of histone deacetylase 1 (HDAC1) is increased in the prefrontal cortex of SCZ subjects, and the mRNA level of its candidate gene GAD1 is decreased (Sharma et al., 2008). Further studies of mouse models have shown that overexpression of HDAC1 in the prefrontal cortex leads to cognitive impairments such as severe impairment of working memory, increased repetitive behaviors, and abnormal motor response profiles in novel environments (Jakovcevski et al., 2013). Unlike HDAC1, PET neuroimaging and other postmortem studies have confirmed that the expression of HDAC2 is decreased in the dorsolateral prefrontal cortex of SCZ patients, which may be associated with cognitive impairment (Schroeder et al., 2017; Gilbert et al., 2019). However, in contrast to the human studies, overexpression of HDAC2 in mice reduced dendritic spine density, synaptic number, synaptic plasticity, and memory formation by inhibiting neuronal gene expression, such as NMDAR subunits (Guan et al., 2009). But anyway, it is clear that epigenetic changes in the NMDAR gene contribute to NMDAR dysfunction, which leads to synaptic dysfunction and cognitive deficits in SCZ (Bharadwaj et al., 2014; Snyder and Gao, 2020). It has been suggested that treatment with HDAC inhibitors may provide new therapeutic avenues for cognitive deficits in SCZ, either alone or in combination with existing drug therapies (Day and Sweatt, 2012; Hasan et al., 2013). In the studies of BD, investigators used HDAC-specific radioactive tracers to measure HDAC expression in BD patients. They suggested that reduced expression of HDAC may contribute to the impairment of emotional or cognitive processes in BD patients by altering neuroplasticity (Tseng et al., 2020). In ASD, loss-of-function mutations in JARID1C, an X-linked gene encoding H3K4 demethylase, may also be associated with cognitive impairment, as it regulates autism and cognitive dysfunction genes such as SCN2A, CACNA1H, BDNF, and SLC18A1 (Adegbola et al., 2008; Shen et al., 2014). In addition, social deficits in a mouse model of SHANK3-deficient autism are rescued by HDAC inhibitors and knockdown of HDAC2 in the prefrontal cortex (Qin et al., 2018). In fact, there are numerous cognition-related histone modifications studies of SCZ and ASD, but there are still few histone studies focusing on cognitive impairment in BD and MDD, and studies of the expression of HDAC2 have been inconsistent between human and mice (Peter and Akbarian, 2011; Day and Sweatt, 2012). Reviewing the literature revealed that a decrease in the activity of HDAC2 during early synaptic development leads to a strong facilitation of excitatory synaptic maturation and a modest increase in synaptic number, in contrast, targeting deletion of HDAC2 in mature neurons impairs the maturation of newborn neurons and basal excitatory neurotransmission in the adult brain (Akhtar et al., 2009). Consequently, it is possible that either too much or too little HDAC2 expression may impair cognitive function. The potential of HDAC2 as a therapeutic target may be limited to specific types of diseases and may even be detrimental to other diseases, such as affective disorders, where the promotion of neurogenesis is indicated (Fischer et al., 2010). However, it is important to stress that, the role of target genes that modified by histone in cognitive impairment of psychiatric disorders is clearly established. Therefore, more attention needs to be paid to the expression of histone-modified target genes in the phenotype of mental cognitive impairment.

As an essential epigenetic regulator, it is gradually understood how non-coding RNA (ncRNA) promotes epigenetic processes and how this phenomenon affects cognitive function in psychiatric disorders. The therapeutic potential of non-coding RNA is that they can affect cognition by targeting a large number of genes and signaling pathways simultaneously, such as multiple pathways within a single neuron (Kocerha et al., 2015). In SCZ, researchers measured the expression of multiple miRNAs in patient serum and postmortem brain (Srivastava et al., 2021). SCZ patients with miR-137 risk alleles show more severe negative symptoms and cognitive impairment. Functional validation and prediction targets for miR-137 include multiple risk genes for cognitive impairment in SCZ, such as CACNA1C, GABRA1, GRIN2A, and HTR2C (Green et al., 2013; Wright et al., 2013). In addition, miR-219 and miR-132 regulate NMDAR signaling, which contributes to some of the neurobehavioral and cognitive impairments seen in SCZ (Kocerha et al., 2009; Miller et al., 2012; Balu et al., 2013). MiR-195 is also increased in the brains of SCZ patients and regulates several cognition-related genes, including BDNF and HTR2A (Pan et al., 2021). In the plasma from BD patients treated with lithium or valproate, which significantly modulate levels of BDNF, a key regulator of neuronal homeostasis, miR-134 is altered and manic symptoms are reduced (Rong et al., 2011). MiR-146a-5p is the strongest candidate for ASD pathology. It is not only the unique miRNA significantly upregulated in the ASD patient cohort and four different cell types, but also its target genes, such as NOTCH1, GRIA3, SYT1, NLGN1, etc., are critical for brains development and function (Mor et al., 2015; Fregeac et al., 2016; Nguyen et al., 2016). In the study of depression, some scholars have demonstrated that the up-regulated expression level of miR-192-5p inhibits the activation of TGF-β1 signaling pathway by binding to Fbln2, thereby improving cognitive impairment and strengthening neurological function in the mouse model of depression (Tang et al., 2019). Overall, the role of miRNAs in cognitive impairment in psychiatric disorders is still under exploration, and similar to methylation and histone modifications, their target molecules are the focus of research. Most of the miRNA changes are directly derived from the postmortem brain and peripheral tissues of patients, reflecting the real situation in the patient’s body, and it also has pleiotropic therapeutic potential. Therefore, it is promising to use miRNA as an objective diagnostic biomarker and a new therapeutic tool for cognitive impairment. However, it is precisely because miRNA has multiple targets that its diagnostic specificity in psychiatric disorders, especially cognitive disorders, remains to be determined. In addition, as a part of an epigenetic network, miRNAs may also be coordinated and regulated by other factors. In the future, we should focus far more on the understanding of ncRNA-mediated epigenetic regulation on well-defined cognitive functions, in order to improve the specificity of its diagnosis and treatment.

## Neurotransmitter deficits

The neurotransmitter system, the basis of cognitive control in humans, has been shown to be associated with a variety of psychiatric disorders. Neurotransmitters involved in cognitive impairment in psychiatric disorders include the glutamatergic system, the dopaminergic neurotransmitter system, gamma-aminobutyric acid (GABA) system, and serotonin system (Wang et al., 2019; Cieślik et al., 2021).

Glutamate synapses transmit most of the fast excitatory neurotransmission and are one of the most critical signals controlling cognitive impairment in psychiatric disorders. Normally glutamate exerts its physiological effects through glutamate-gated ion channels (ionotropic glutamate receptors, iGluRs) and glutamate-activated G protein-coupled receptors (metabotropic glutamate receptors, mGluRs) (Volk et al., 2015). Among them, the ongoing hypofunction of glutamatergic NMDAR is considered to be a key factor of cognitive impairment (Javitt et al., 2018). In SCZ, multiple lines of evidence in pharmacological, genetic, neuroimaging, and postmortem studies support that NMDAR hypofunction may underlie the symptoms of cognitive impairment (Corlett et al., 2011; Moghaddam and Javitt, 2012; Schizophrenia Working Group of the Psychiatric Genomics Consortium, 2014; Kort et al., 2017). Abnormalities of NMDAR-mediated neurotransmission have been implicated in behavioral, social and cognitive deficits of ASD associated with a variety of risk alleles (Liu et al., 2017; Wang et al., 2018; Deutsch et al., 2022). Positive allosteric modulators (PAMs) of NMDAR are also used to treat cognitive deficits in SCZ and some forms of autism (Paul et al., 2013; Burnell et al., 2019). But in studies related to depression, the NMDAR antagonist Memantine improved symptoms of anhedonia and anxiety, as well as cognitive deficits associated with depression (Li et al., 2020).

The dopaminergic system, a key neurotransmitter system, are broadly implicated in psychiatric illnesses (Grace, 2016). Dopamine receptors in the brain are mainly divided into two categories with opposite roles: excitatory D1-like receptors (D1R, include D1R and D5R) and inhibitory D2-like receptors (D2R, including D2R, D3R, and D4R) (Wei et al., 2018). The traditional view of psychiatric disorders claims that positive symptoms are associated with hyperdopaminergic activity and mediated by D2R, while deficits in D1R-mediated dopamine activity are responsible for negative symptoms and cognitive impairment (Lindenmayer et al., 2013; Locke et al., 2018). Therefore, D1R agonists are used to improving cognitive impairment, such as the development of agonists for functionally selective D1 ligands and positive allosteric modulators of D1R (Arnsten et al., 2017; Desai et al., 2021; Abi-Dargham et al., 2022). D2R have also received considerable attention in recent years as regulators of cognitive impairment. Dopamine D3R plays a functional role in performing new object recognition tasks through GSK3β signaling in medial prefrontal cortex. Therefore, D3 R antagonist therapy may be a reasonable method to improve cognitive impairment or episodic memory deficit in BD patients (Chang et al., 2020). In the treatment of SCZ, a highly potent preferential D3 antagonist, F17464, is also being developed, which can significantly improve cognitive function (Bitter et al., 2019). Moreover, recent studies prove that stress exposure in dopamine D4R knockout mice induced significant deficits in SCZ-like behaviors, such as sensorimotor gating, cognitive processes, social engagement, and exploratory behavior, by disrupting GABAergic transmission (Tan et al., 2019).

GABA is a major inhibitory neurotransmitter in the mammalian brain. Dysregulated GABA interneuron activity disrupts the excitatory/inhibitory (E/I) balance in the cortex, which may represent the core pathophysiology of cognitive dysfunction in SCZ, mainly includes the following three aspects (Xu and Wong, 2018). First, reduced expression of parvalbumin (PV) and GAD67 has been found to be associated with altered GABA transmission in SCZ (Lewis et al., 2012; Lewis, 2014; Kim et al., 2016). Second, deficits in GABA inhibitory circuits lead to impaired neural oscillations in SCZ (Lewis et al., 2008; Uhlhaas and Singer, 2010; Chen et al., 2014). Third, alterations in other GABA genes related to GABA neurotransmission, such as GABA transporter type 1 (GAT-1) and NMDA receptor subunits (e.g., NR2A and NR3A) (Scheggia et al., 2018; Xu and Wong, 2018). Similarly, evidence from anatomy, as well as from human and rodent genetics, physiology, and behavior demonstrate that altered balance in E/I is often referred to as a possible ultimate common pathway in autism (Selimbeyoglu et al., 2017). Decreased number of PV neurons, decreased expression of mRNA encoding receptors for the inhibitory neurotransmitter GABA, and decreased ratio of GABA to glutamate have also been observed in autism patients and in mouse models (Chao et al., 2010; Fatemi et al., 2010; Alabdali et al., 2014; Hashemi et al., 2017). Moreover, changes in GABAergic neuropathology, such as RELN and GAD67, have been detected in the hippocampus and cortex in the brains of patients with BD (Fatemi et al., 2000; Guidotti et al., 2000; Veldic et al., 2007). Decreased GABA levels in the prefrontal cortex, anterior cingulate cortex, cerebrospinal fluid, and plasma have also been demonstrated in studies of major depression (Klempan et al., 2009; Levinson et al., 2010; Northoff and Sibille, 2014; Fee et al., 2017). In conclusion, given the importance of the GABA system in cognitive impairment in psychiatric disorders, it has emerged as a potential target for new pharmacological interventions. Currently, α5/α3/α2 GABA_*A*_ and GABA_*B*_-mediated pharmacologically modulated receptors have been shown to restore E/I imbalance and cognitive deficits in SCZ patients, and other antagonists, positive and negative allosteric modulators (including dual allosteric modulators), GAT-1 inhibitors, etc., have shown promise in animal preclinical studies (Rudolph and Möhler, 2014; Xu and Wong, 2018; Zafar and Jabeen, 2018). Recent ideas suggest that dual allosteric modulators can reduce the dose required to achieve optimal therapeutic effect, reduce side effects caused by individual drugs, and enhance pro-cognitive effects (Xu and Wong, 2018). Therefore, intentional targeting of multiple receptors may be a promising strategy for improving pharmacological treatment of cognitive impairment in schizophrenia and other neuropsychiatric disorders.

Serotonin (5-hydroxytryptamine, 5-HT) is a monoamine neurotransmitter that regulates central and peripheral functions and is involved in emotional and appetite regulation, cognition and memory (Meneses, 1999). Increased 5-HT signaling is thought to be associated with SCZ and ASD (Sumiyoshi et al., 1996; Selvaraj et al., 2014; Chen et al., 2017), while decreased 5-HT signaling is thought to be associated with MDD and BD (Lemonde et al., 2003; Albert and Lemonde, 2004). Partial antipsychotic agonists and antagonists targeting the 5-HT 2A receptor and the 5-HT6 receptor have shown some beneficial antipsychotic and pro-cognitive effects in SCZ and ASD (Meltzer et al., 2003; Amodeo et al., 2014; de Bruin and Kruse, 2015; Fung et al., 2016; Keefe et al., 2018; Yamazaki et al., 2018). Selective serotonin reuptake inhibitors (SSRIs) are novel types of antidepressants, which can inhibit the reuptake of serotonin at synapses, thereby increasing the content of serotonin in the brain and improving cognitive function in patients with MDD, especially vortioxetine, which also modulates 5-HT receptors (Frampton, 2016; Liu L. et al., 2021). In addition to these systems, the cholinergic system is also used to find and develop treatments for cognitive impairment in SCZ. Still, the precise nature of the cholinergic disorder in patients and its impact on cognitive functions, including attention, is poorly understood to date (Sarter et al., 2012; Santos et al., 2018; Recio-Barbero et al., 2021).

The above studies and summaries show that different neurotransmitter systems play different roles in cognitive impairment. Detailed neurotransmitter systems associated with cognitive impairment in psychiatric disorders was summarized in Supplementary Table 1. Currently, the main specific use of the glutamate system in SCZ-related cognitive impairment is represented by the Glycine transporter-1 (GlyT1) inhibitor BI 425809, which enhances NMDAR function through GlyT1 inhibitor and has been granted breakthrough therapy designation by the US Food and Drug Administration (Fleischhacker et al., 2021; Veselinović and Neuner, 2022). In addition, Vortioxetine, the 5-HT system drug, is an FDA-approved multimodal antidepressant that uniquely improves cognitive impairment associated with major depression (Liu L. et al., 2021). Cognitive impairment compounds targeting other systems are still in preclinical animal studies or late development, requiring large samples and long-term individualized studies to obtain effective strategies.

## Proinflammatory cytokines and neurotrophins

Peripheral blood derived biomarkers, which are combined with easy and minimally invasive accessibility, provide a more acceptable and inexpensive option for the diagnosis, monitoring and prognosis of cognitive impairment in mental diseases in recent years. As the representative molecules of inflammation, interleukin-6 (IL-6), IL-1β, tumor necrosis factor alpha (TNF-α), and C-reactive protein (CRP) in peripheral blood of patients with SCZ, BD, ASD, and MDD have received considerable attention.

IL-6 and TNF-α in SCZ are reported to be positively correlated with poor cognitive performance in most studies, and are often used to evaluate the efficacy of pro-cognitive compounds (Frydecka et al., 2015; Kogan et al., 2018; Zhang et al., 2019; Zhu et al., 2019; King et al., 2021; Adamowicz et al., 2022). The neurocognitive role of IL-6 in SCZ may be due to its ability to alter the survival and transmission of catecholaminergic, serotonergic, and dopaminergic neurons in the hippocampus and prefrontal cortex (Zalcman et al., 1994; Frydecka et al., 2015). There is also evidence that IL-6 increases choroid plexus volume, thinning gray matter and shrinking amygdala, changes that may ultimately lead to cognitive decline (Lizano et al., 2019). Similar studies have shown that high levels of IL-6 in BD, MDD, and ASD exhibit symptoms of neurocognitive deficits, such as impaired sustained attention and orientation and short-term memory (Wei et al., 2011; Barbosa et al., 2018; Jin et al., 2020; Nie et al., 2021).

TNF-α plays a vital role in brain development as it regulates synaptic pruning and plasticity in preadolescence, adolescence, and early adulthood, mediating neuronal homeostasis and dysfunctional signaling (Stellwagen and Malenka, 2006; Park and Bowers, 2010). Studies have shown that levels of soluble tumor necrosis factor receptor 2 (sTNFR2) in plasma are associated with hippocampal volume and cognitive performance in SCZ (Kudo et al., 2018). Other studies have reported TNF variables during the neural progression of BD, suggesting that circulating levels of TNF molecules partially mediate the relationship between prior severe mood episodes and executive functioning in BD, and may serve as novel cognitive interventions (Millett et al., 2020; Zazula et al., 2022). Circadian dysregulation in ASD is strongly associated with elevated TNF levels and immune-inflammatory activity, leading to sleep disturbances and cognitive and behavioral changes (da Silveira Cruz-Machado et al., 2021). TNF-α antagonism has provided encouraging findings in improving depressive symptoms in chronic inflammatory diseases, but direct studies of TNF-α in cognitive impairment in MDD are lacking (Bortolato et al., 2015). Therefore, we still need to tap into the cognitive potential targets in people with primary depression.

The researches of IL-1β and CRP of cognitive impairment in psychiatric disorders is still controversial. Studies have shown that IL-1β may also be a state marker of acute exacerbation in SCZ (Miller et al., 2011). In addition, peripheral IL-1β mRNA levels were found to be negatively correlated with verbal fluency and Broca’s area volume in patients with SCZ, and this study linked inflammatory blood biomarkers to cognitive deficits and reduced brain volume in SCZ patients (Fillman et al., 2016). However, other studies have suggested that peripheral IL-1β has no effect on neurocognitive and daily functioning in patients with SCZ (Kogan et al., 2018). In studies of patients hospitalized for bipolar depressive episodes, higher levels of IL-1β were associated with decreased cognitive performance (Poletti et al., 2021). The negative correlation between IL-1β level and overall cognitive function was also observed in MDD patients (Jin et al., 2020).

C-reactive protein is an extensively studied inflammatory biomarker. Some studies suggest that the level of serum high sensitivity C-reactive protein (hs-CRP) is positively correlated with cognitive impairment in patients with SCZ, such as executive function and processing speed, and may represent an objective biological indicator for rapid assessment of cognitive impairment in patients with SCZ (Misiak et al., 2018; Bora, 2019; Zhu et al., 2019; Adamowicz et al., 2022). However, contrary views suggest that hs-CRP is not associated with cognitive impairment, and that higher hs-CRP levels in SCZ may be associated with female and more severe negative symptoms (Joseph et al., 2015). In studies of BD, elevated levels of CRP inflammation may be associated with a broad range of cognitive deficits in patients, and researches have also identified CRP as a significant positive predictor of proxy cognitive decline (Dickerson et al., 2013; Millett et al., 2021).

Neurotrophins, particularly BDNF and nerve growth factor (NGF), are another type of cytokines associated with cognitive impairment in psychiatric disorders. BDNF and NGF regulate neuronal survival and growth, and affects synaptic efficiency and plasticity (Sofroniew et al., 2001; Park and Poo, 2013). Although several studies have confirmed that BDNF and NGF are expressed at lower levels in peripheral blood of patients with SCZ, MDD, and BD, their association with cognitive impairment remains controversial (Green et al., 2011; Qin et al., 2017; Rao et al., 2017). Some suggest that reduced BDNF levels do not play a major role in the cognitive dysfunction of most SCZ and BD patients (Dias et al., 2009; Bora, 2019). However, recent studies have shown that low serum BDNF levels are associated with cognitive impairment in patients with first-episode and chronic SCZ and non-medicated patients with current depressive episodes of BD II and MDD (Yang et al., 2019; Teng et al., 2021).

Other reported inflammatory biomarker complexes most associated with SCZ cognition include intercellular adhesion molecule-1 and serum amyloid protein (Adamowicz et al., 2022). Inflammatory chemokines associated with cognitive deficits in BD patients include CCL2, CCL4, CCL5, CXCL10, and basic fibroblast growth factor (bFGF) (Poletti et al., 2021).

We note that a recent systematic review and meta-analysis of immune markers in surface blood showed only weak associations with perceptions of mood and psychiatric disorders (SCZ, BD, and MDD), the investigators consider that the observed significant publication and reporting bias is likely to underlie such association inflation in individual studies (Morrens et al., 2022). However, we cannot completely deny the role of peripheral cytokine markers. Table 1 summarizes the key proinflammatory cytokines and neurotrophins associated with cognitive impairment in psychiatric disorders. The number of inflammatory cytokines is indeed associated with an increased risk of psychosis, most notably IL-1β, IL-6, TNF-α, CRP, and BDNF, although the current literature remains inconsistent (Kogan et al., 2020). More detailed assessments and advanced detection methods are still needed to exploit the value of peripheral biomarkers in future clinical applications.

**TABLE 1:** Peripheral inflammatory factors and neurotrophic factors associated with cognitive impairment in psychiatric disorders.

Cytokines	Description	Disease	References
IL-6	The neurocognitive role of IL-6 in SCZ may be due to its ability to alter the survival and transmission of catecholaminergic, serotonergic, and dopaminergic neurons in the hippocampus and prefrontal cortex	BD, MDD, and ASD	Zalcman et al., 1994; Wei et al., 2011; Frydecka et al., 2015; Barbosa et al., 2018; Jin et al., 2020; Nie et al., 2021
TNF-α	Levels of sTNFR2 in plasma are associated with hippocampal volume and cognitive performance	SCZ, BD, and ASD	Kudo et al., 2018; Millett et al., 2020; da Silveira Cruz-Machado et al., 2021; Zazula et al., 2022
IL-1β	Peripheral IL-1β mRNA levels were found to be negatively correlated with verbal fluency and Broca’s area volume	SCZ and BD	Fillman et al., 2016; Poletti et al., 2021
CRP	The level of serum hs-CRP is positively correlated with cognitive impairment such as executive function and processing speed	SCZ and BD	Misiak et al., 2018; Bora, 2019; Zhu et al., 2019; Adamowicz et al., 2022
BDNF and NGF	BDNF and NGF regulate neuronal survival and growth, and affects synaptic efficiency and plasticity	SCZ, BD, and MDD	Sofroniew et al., 2001; Park and Poo, 2013; Yang et al., 2019; Teng et al., 2021
Intercellular adhesion molecule-1 and serum amyloid protein	They are best correlated with scores on cognitive testing	SCZ	Adamowicz et al., 2022
IL-1β, IL-6, CCL2, CCL4,CCL5,CXCL-10, and bFGF	Higher levels of IL-1β, IL-6, CCL2, CCL4, CCL5, CXCL10, and bFGF are associated with the likelihood of having a poor cognitive performance	BD	Poletti et al., 2021

## Metabolism and microbiome-gut-brain axis

Of note, a growing body of evidence in recent years has indicated that metabolic mechanisms have been linked to learning and memory, mental and emotional health, and neuropsychiatric disorders (Sánchez-Ortí et al., 2022). Several common aspects have been implicated, including glucose metabolism, lipid metabolism, and microbiome-gut-brain axis.

A study of glucose disorders, cognitive deficits, and white matter abnormalities in first-episode drug-free SCZ has raised strong concerns, suggesting that the interaction between abnormal glucose metabolism and white matter disconnectivity may contribute to cognitive impairment (Zhang X. et al., 2020). In the same year, the role of glycosylation in SCZ was revealed by GWAS (Mealer et al., 2020). Other researchers have also highlighted that proteins involved in excitatory and inhibitory neurotransmission show altered glycan effects in disease states, including α-amino-3-hydroxy-5-methyl-4-isoxazolpropionic acid (AMPA) and kainate receptor subunits, glutamate transporters excitatory amino acid transporters 1 and 2, and γ-aminobutyric acid A (GABAA) receptors, etc. (Williams et al., 2020). Indeed, two meta-analysis have shown evidence of a significant relationship between cognitive impairment in SCZ and each component of the metabolic syndrome, including hypertension, dyslipidemia, abdominal obesity, and diabetes (Bora et al., 2017; Hagi et al., 2021). Cognitive dysfunction in SCZ may also be associated with central insulin deficits, which involve the regulation of dopamine, glucose metabolism and feeding, and exercise or pharmacological interventions to improve insulin sensitivity may be one way to address cognitive deficits in SCZ (Li et al., 2014; Agarwal et al., 2020). Impaired frontolimbic circuits are associated with significantly reduced glucose uptake, which may account for reduced executive function in BD-I patients during remission (Li et al., 2012). In addition, insulin resistance was significantly associated with verbal memory performance in remitting BD participants without diabetes, even after controlling for other relevant metabolic or therapeutic variables (Salvi et al., 2020). Recent studies also suggest that elevated levels of fasting blood glucose and triglyceride may be associated with cognitive dysfunction in BD patients. Improving glucose and lipid metabolism in BD patients may help improve some specific areas of cognitive function (Qiu et al., 2022). In ASD subjects, the rate of glucose metabolism in parietal, prefrontal motor and eye areas and amygdala was decreased, which may be related to cognitive tasks (Mitelman et al., 2018). A study of the relationship between insulin resistance and cognition in patients with depression found that higher steady-state plasma glucose was associated with poorer cognitive flexibility in young adults (Wroolie et al., 2015).

Many studies have attempted to clarify the association between cognitive impairment and lipid metabolism in psychiatric disorders. An interesting study found that specific erythrocyte membrane lipid clusters are associated with clinical and cognitive manifestations of dopamine dysfunction in SCZ and hypothesized that this membrane lipid abnormality affects presynaptic dopamine signaling (Tessier et al., 2016). Polyunsaturated fatty acids (PUFA), especially ω-3PUFAs, have also been described as important modulators that affect brain resilience and thus central nervous system function (Wysoczański et al., 2016). ω-3PUFAs can ameliorate cognitive impairment by upregulating BDNF in SCZ to repair neuronal damage (Guo et al., 2020; Tang et al., 2020). In patients with BD and SCZ, alterations of apolipoprotein C, which affects lipid metabolism and utilization, result in a decline in cognitive performance and hippocampal volume (Knöchel et al., 2017). Studies of BD have also shown that reduced high-density lipoprotein and elevated triglycerides (TG) levels are associated with cognitive deficits, especially in the immediate memory, language domains and executive functioning in BD patients (Hui et al., 2019; Van Rheenen et al., 2021). Consistently, high serum TG levels showed poor cognitive function in MDD, especially in memory delay (Shao et al., 2017; Guan et al., 2021).

The gut microbiome is a complex microbial ecosystem that has been clearly shown to influence host psychological, emotional, and cognitive functions through various neurophysiological aspects of synaptic plasticity, and may be a key regulator of neural development of the microbiome-gut-brain axis (Rogers et al., 2016; Glinert et al., 2022). Fecal transfer of gut microbiota from SCZ patients has been reported to induce SCZ-related behaviors in germ-free recipient mice, accompanied by alterations in levels of glutamate, glutamine and GABA in the hippocampus (Zheng et al., 2019). Further studies of the metagenome-wide association of gut microbiome features for SCZ identified 27 gut-brain modules, whose bacterial functional potentials include differences in short-chain fatty acid synthesis, tryptophan metabolism, and neurotransmitter synthesis/degradation. In addition, transplanting SCZ-enriched bacterium, Streptococcus vestibularis, appears to lead to social behavioral deficits and alter neurotransmitter levels in peripheral tissues in the recipient mice (Zhu et al., 2020). It has also been found that BD-related microbiota, such as Citrobacter spp. and Phascolarctobacterium spp., can synthesize corresponding neuroactive metabolites, which are potential markers associated with hallmarks of functional connectivity in brain networks, and imply abnormal cognitive function, emotion regulation, and interoception (Li et al., 2022). In studies of ASD, when gut microbiota from human donors with ASD were transplanted into germ-free mice, their brains showed alternative splicing of ASD-related genes that induced signature autistic behaviors. The researchers also treated a mouse model of ASD with candidate microbial metabolites and found that the behavioral abnormalities were improved and neuronal excitability were modulated in the mice (Sharon et al., 2019). Interestingly, a new research suggests that autism-related dietary preferences mediate the autism-gut microbiome association, rather than the microbiome driving ASD (Yap et al., 2021). Studies on MDD have shown that the increase of some proinflammatory bacteria and the decrease of some anti-inflammatory bacteria in patients are related to inflammatory factors (IL-1β, IL-6, and TNF-α) and cognitive function, and intestinal microbiota may be responsible for cognitive impairment in patients with MDD (Liu P. et al., 2021).

On the whole, the importance of metabolic syndrome, including obesity, overweight, hypertension, dyslipidemia and insulin resistance, and the microbiome-gut brain-axis in cognitive impairment is gradually being recognized. Researchers have proposed four main therapeutic strategies based on these: wild-type and transgenic probiotics, fecal microbiota transplantation, physical exercise, and a high-fiber diet, and link these therapies to inflammation reduction. These strategies have the potential to be one of the most important areas in the treatment prospects of cognitive impairment in mental illness (Sun et al., 2020).

## Stem cell therapies

As a therapeutic tool for regenerative medicine, stem cells provide new insights into the treatment of neuropsychiatric disorders and cognitive deficits. Common sources include induced pluripotent stem cells (iPSCs), mesenchymal stem cells (MSCs), and neural stem cells (NSCs), which are considered suitable types for transplantation into various animal models with neuropsychopathology and cognitive disorders (Zhang Y. et al., 2020).

Reprogrammed iPSCs from patient-derived somatic cells contribute significantly to recapitulating the development of neuronal cells with disease-specific genetic backgrounds (Soliman et al., 2017). Modeling techniques for inducing disease in iPSCs, including directed differentiation of neuronal subtypes, glial cells, and 3D brain organoid models, are handy for modeling how complex genetic variation leads to disease pathogenesis (Wang et al., 2020; Whiteley et al., 2022). The researchers directly reprogrammed human induced pluripotent stem cells iPSCs from SCZ patients, and then differentiated these disease-specific iPSCs into neurons. SCZ iPSCs neurons showed decreased neuronal connectivity, which was associated with decreased neurite number, PSD95 protein level, and glutamate receptor expression. They also evaluated several antipsychotics on neuronal cultures derived from iPSCs from SCZ patients to assess their ability to rescue neuronal connectivity deficits and other phenotypes found in SCZ neurons (Brennand et al., 2011). In studies of iPSCs-derived brain organoids in SCZ, brain organoids from SCZ patients showed aberrant gene expression in pathways involved in synaptic biology, neurological development, immune responses, and mitochondrial function, as well as specific defects in mitochondrial physiology and diminished responses to stimulation and depolarization (Kathuria et al., 2020a). Similar studies in brain organoids from BD patients have shown abnormalities in the regulation of genes involved in cell adhesion, immune signaling, and ER biology, as well as evidence of defects in neurotransmission (Kathuria et al., 2020b). IPSCs-derived neurons from patients with Rett syndrome (a genetic model of ASD) also exhibit morphological abnormalities, such as fewer synapses, reduced spine density, smaller somatic cell size, altered calcium signaling, and electrophysiological defects, which may be related to GABA dysfunction (Marchetto et al., 2010). Serotonin neurons are dysregulated in depression and are commonly targeted by antidepressants. What is more exciting, two independent research groups have now greatly advanced iPSC research by generating serotonergic neurons that can be studied *in vitro*, thus potentially bringing them into cognitive impairment studies of depression (O’Shea and McInnis, 2016; Vadodaria et al., 2016; Xu et al., 2016).

Mesenchymal stem cells are one of the most promising stem cell types in therapeutic strategies due to their potent immunomodulatory activity and paracrine regeneration mechanism making them relevant to various diseases related to inflammation and tissue damage (Liu et al., 2019). Intranasal administration of MSC-derived exosomes improved the disruption of social interaction and prepulse inhibition in phencyclidine treated mice by maintaining the viability of GABA-producing neurons, thus significantly attenuating SCZ-like behavior (Tsivion-Visbord et al., 2020). Human umbilical cord MSCs can induce the regulation and production of T peripheral anti-inflammatory cytokine IL-10 and attenuate SCZ related social deficit behaviors in amphetamine-sensitized mice (You et al., 2020). In addition, intraventricular transplantation of bone marrow MSCs also enhanced hippocampal neurogenesis in a mouse model of ketamine-induced neurodevelopment of SCZ, thereby improving social novelty preference (Gobshtis et al., 2021). MSCs have also demonstrated therapeutic potential for immune dysfunction in the pathogenesis of ASD (Liu et al., 2019). In mouse studies with ASD, MSCs transplantation reduced stereotypic behavior, cognitive rigidity, and improved social behavior by increasing hippocampal BDNF protein levels and neurogenesis (Segal-Gavish et al., 2016; Perets et al., 2017). ASD mice treated with intranasal administration of the medicinal MSCs-exosome showed significant improvements in social domains, ultrasonic communication, and repetitive behaviors (Perets et al., 2018). Multiple clinical trials have reported favorable safety and efficacy of MSCs transplantation in children with ASD, including improvements in social relationships and reciprocity, emotional reactivity, speech, language, communication, behavioral patterns, sensory aspects, and cognition (Lv et al., 2013; Sharma et al., 2013). Moreover, the combination of cord blood-MSCs and cord blood monocytes also significantly improved the symptom severity of ASD (Lv et al., 2013). Studies have also shown that adipose-derived MSCs protect mice from chronic mild stress-induced depression-like behaviors by modulating Nrf2/HO-1 and TLR4/NF-κB signaling pathways, therefore, the role of MSCs in the treatment of cognitive impairment in MDD is also anticipated (Huang et al., 2020).

Neural stem cells are known to be essential for brain development and postnatal maintenance of neurogenesis in specific brain regions (Bertolini et al., 2019). Studies from NSCs in the biological chemical purification the transcriptional regulatory factors observed that transcription regulatory factors associated with ASD or SCZ are part of the neural stem cell transcriptional network. The network of transcription factor protein mutations is associated with mental disorders, including low IQ, suggesting that shared transcription network damage level is associated with cognitive dysfunction (Moen et al., 2017).

These studies demonstrate that these three types of stem cells have broad clinical application prospects. IPSCs technology offers new opportunities to mimic disease-related nerve cells from patients (Wang et al., 2020). MSCs and their exosomes, including miRNA, nutrient factors, enzymes and immune regulation derived from them, have shown advantages in the treatment of cognitive disorders (Harrell et al., 2021). The severity of mutations in NSCs-associated transcription factors network proteins highlights a close correlation with the level of cognitive dysfunction (Moen et al., 2017). However, paradoxically, the effectiveness and safety of stem cells almost simultaneously exist, which is a dilemma we must face. These concerns make the clinical application of stem cells limited by safety and ethical issues. Even more exciting for us, with innovations in stem cell disease modeling methods, and precise disease-specific therapeutic doses, regimens, and pathways, new technologies pave the way for future personalized medicine research on stem cells and more effective drug discovery processes in cognitive disorders (Whiteley et al., 2022).

## Conclusion

In this review, we discuss in detail the mechanisms of cognitive impairment in psychiatric disorders including SCZ, BD, ASD, and MDD. An overview of this review is shown in Figure 1. (i) Genetics relies on variation and allele frequency data to infer essential risk genes and population genetic analyses for cognitive function; (ii) Epigenetics complements the mechanistic link between environmental exposures and the expression profiles of genes in cognitive impairment; (iii) Defects and imbalances in neurotransmitter systems underlie cognitive impairment in most psychiatric disorders; (iv) Peripheral inflammatory factors and neurotrophic factors are key biomarkers of cognitive impairment in psychiatric disorders; (v) The metabolism and microbial gut-brain axis are critical bidirectional communication systems in cognitive impairment in psychiatric disorders; and (vi) Stem cells provide an efficient *in vitro* modeling and therapeutic approach for the mechanism research of cognitive impairment in mental illness.

Although we have discussed the molecular mechanisms of cognitive impairment in several different ways, they are not completely separate. In fact, the different signals may interact with each other, which contributes to the cognitive impairment of mental illness. Defects in various neurotransmitters and abnormal expression of cytokines constitute the effector molecules and basis of cognitive phenotype. Significant neurotransmitters include NMDAR, dopamine receptors, GABA, and 5-HT (Wang et al., 2019; Cieślik et al., 2021). Common cytokines include IL-1β, IL-6, TNF-α, CRP, BDNF, and NGF (Park and Poo, 2013; Kogan et al., 2020). Genetics have identified certain susceptibility loci, including neurotransmitter (e.g., NMDA and NMDAR) and cytokine-related genes (e.g., NRG3), to be significantly associated with cognitive impairment (Ohi et al., 2015; Greenwood et al., 2019). Epigenetics and genetics cooperate to control genome activity in cognitive disorders. Both the target genes of DNA methylation, histone modification, and non-coding RNAs are also various neurotransmitters and cytokines. The genes of cognitive impairment modified by DNA methylation include GAD1, BDNF, DRD3, DRD4, and 5HTR1A (Dong et al., 2005; Carrard et al., 2011; Chen et al., 2014; Dai et al., 2014). The genes of cognitive impairment modified by histone include GAD1, GAD2, NMDAR, CACNA1H, and BDNF (Huang et al., 2007; Adegbola et al., 2008; Snyder and Gao, 2020). The genes of cognitive impairment regulated by non-coding RNAs include GRIN2A, BDNF, HTR2A, and NMDAR (Balu et al., 2013; Wright et al., 2013; Pan et al., 2021). Moreover, metabolism and microbiome-gut-brain axis link gut and brain activities, and they function through various neurotransmitters and cytokines as well, such as GABA, IL-1β, IL-6, and TNF-α (Zheng et al., 2019; Liu P. et al., 2021). Stem cells provided a valuable tool for modeling techniques and potential therapeutic applications (Zhang Y. et al., 2020). They can ameliorate cognitive impairment through paracrine or immunomodulatory effects, and their effector molecules are also neurotransmitters and cytokines, such as GABA, IL-10, and BDNF (Perets et al., 2017; Tsivion-Visbord et al., 2020; You et al., 2020). These mechanisms involve multiple processes of gene expression and regulation, from gene level, transcriptional level, post-transcriptional level to actual expression *in vivo*. In summary, our review provides some kind of complete and dynamic approach to the analysis of the cognitive impairment phenotype of psychiatric disorders.

The specific strengths and weaknesses of each mechanism for diagnosing, treating, and preventing cognitive impairment in mental illness are also reviewed in each section. Based on the current review, we conclude that genetics, epigenetics, and neurotransmitter systems, inflammatory factors, and neurotrophic factors seem to be the more reliable diagnostic basis for cognitive impairment in psychiatric disorders. Moreover, the neurotransmitter system, metabolic and microbial gut-brain axis, and stem cells may be effective targets and tools for the treatment and prevention of cognitive impairment in psychiatric disorders.

Finally, the shortcomings mentioned above in the diagnosis, treatment, and prevention mechanisms remain challenges and opportunities that we will continue to face in the future. With rapid advancements in genomics, molecular biology, materials science, innovative technologies, and research methods, biomarkers for various cognitive impairment mechanisms in psychiatric disorders will have great potential for accuracy, sensitivity, and specificity for future diagnosis and treatment.

## Author contributions

YW and WM wrote the first draft of the manuscript. ZL searched the references and revised the manuscript. QA and XH designed the outline and approved the final draft of the manuscript. All authors contributed to the article and approved the submitted version.

## References

[B1] Abdolmaleky H. M. Nohesara S. Ghadirivasfi M. Lambert A. W. Ahmadkhaniha H. Ozturk S. (2014). DNA hypermethylation of serotonin transporter gene promoter in drug naïve patients with schizophrenia. *Schizophr. Res.* 152 373–380. 10.1016/j.schres.2013.12.007 24411530 PMC7863587

[B2] Abi-Dargham A. Javitch J. A. Slifstein M. Anticevic A. Calkins M. E. Cho Y. T. (2022). Dopamine D1R receptor stimulation as a mechanistic pro-cognitive target for schizophrenia. *Schizophr. Bull.* 48 199–210. 10.1093/schbul/sbab095 34423843 PMC8781338

[B3] Adamowicz D. H. Shilling P. D. Palmer B. W. Nguyen T. T. Wang E. Liu C. (2022). Associations between inflammatory marker profiles and neurocognitive functioning in people with schizophrenia and non-psychiatric comparison subjects. *J. Psychiatr. Res.* 149 106–113. 10.1016/j.jpsychires.2022.02.029 35259663 PMC9933244

[B4] Adegbola A. Gao H. Sommer S. Browning M. (2008). A novel mutation in JARID1C/SMCX in a patient with autism spectrum disorder (ASD). *Am. J. Med. Genet. A* 146A 505–511. 10.1002/ajmg.a.32142 18203167

[B5] Agarwal S. M. Caravaggio F. Costa-Dookhan K. A. Castellani L. Kowalchuk C. Asgariroozbehani R. (2020). Brain insulin action in schizophrenia: Something borrowed and something new. *Neuropharmacology* 163:107633. 10.1016/j.neuropharm.2019.05.010 31077731

[B6] Akbarian S. Liu C. Knowles J. A. Vaccarino F. M. Farnham P. J. Crawford G. E. (2015). The PsychENCODE project. *Nat. Neurosci.* 18 1707–1712. 10.1038/nn.4156 26605881 PMC4675669

[B7] Akhtar M. W. Raingo J. Nelson E. D. Montgomery R. L. Olson E. N. Kavalali E. T. (2009). Histone deacetylases 1 and 2 form a developmental switch that controls excitatory synapse maturation and function. *J. Neurosci.* 29 8288–8297. 10.1523/jneurosci.0097-09.2009 19553468 PMC2895817

[B8] Alabdali A. Al-Ayadhi L. El-Ansary A. (2014). Association of social and cognitive impairment and biomarkers in autism spectrum disorders. *J. Neuroinflammation.* 11:4. 10.1186/1742-2094-11-4 24400970 PMC3896747

[B9] Albert P. R. Lemonde S. (2004). 5-HT1A receptors, gene repression, and depression: Guilt by association. *Neuroscientist* 10 575–593. 10.1177/1073858404267382 15534042

[B10] Amodeo D. A. Jones J. H. Sweeney J. A. Ragozzino M. E. (2014). Risperidone and the 5-HT2A receptor antagonist M100907 improve probabilistic reversal learning in BTBR T + tf/J mice. *Autism Res.* 7 555–567. 10.1002/aur.1395 24894823 PMC4230321

[B11] Arnsten A. F. Girgis R. R. Gray D. L. Mailman R. B. (2017). Novel dopamine therapeutics for cognitive deficits in schizophrenia. *Biol. Psychiatry* 81 67–77. 10.1016/j.biopsych.2015.12.028 26946382 PMC4949134

[B12] Balu D. T. Li Y. Puhl M. D. Benneyworth M. A. Basu A. C. Takagi S. (2013). Multiple risk pathways for schizophrenia converge in serine racemase knockout mice, a mouse model of NMDA receptor hypofunction. *Proc. Natl. Acad. Sci. U.S.A.* 110:E2400–E2409. 10.1073/pnas.1304308110 23729812 PMC3696825

[B13] Barbosa I. G. Ferreira R. A. Rocha N. P. Mol G. C. da Mata Chiaccjio Leite F. Bauer I. E. (2018). Predictors of cognitive performance in bipolar disorder: The role of educational degree and inflammatory markers. *J. Psychiatr. Res.* 106 31–37. 10.1016/j.jpsychires.2018.09.003 30261412

[B14] Bertolini J. A. Favaro R. Zhu Y. Pagin M. Ngan C. Y. Wong C. H. (2019). Mapping the global chromatin connectivity network for sox2 function in neural stem cell maintenance. *Cell Stem Cell* 24 462–476.e6. 10.1016/j.stem.2019.02.004 30849367 PMC6506828

[B15] Bharadwaj R. Peter C. J. Jiang Y. Roussos P. Vogel-Ciernia A. Shen E. Y. (2014). Conserved higher-order chromatin regulates NMDA receptor gene expression and cognition. *Neuron* 84 997–1008. 10.1016/j.neuron.2014.10.032 25467983 PMC4258154

[B16] Bitter I. Lieberman J. A. Gaudoux F. Sokoloff P. Groc M. Chavda R. (2019). Randomized, double-blind, placebo-controlled study of F17464, a preferential D(3) antagonist, in the treatment of acute exacerbation of schizophrenia. *Neuropsychopharmacology* 44 1917–1924. 10.1038/s41386-019-0355-2 30822774 PMC6785149

[B17] Bora E. (2019). Peripheral inflammatory and neurotrophic biomarkers of cognitive impairment in schizophrenia: A meta-analysis. *Psychol. Med.* 49 1971–1979. 10.1017/s0033291719001685 31284882

[B18] Bora E. Akdede B. B. Alptekin K. (2017). The relationship between cognitive impairment in schizophrenia and metabolic syndrome: A systematic review and meta-analysis. *Psychol. Med.* 47 1030–1040. 10.1017/S0033291716003366 28032535

[B19] Bortolato B. Carvalho A. F. Soczynska J. K. Perini G. I. Mcintyre R. S. (2015). The involvement of TNF-α in cognitive dysfunction associated with major depressive disorder: An opportunity for domain specific treatments. *Curr. Neuropharmacol.* 13 558–576. 10.2174/1570159x13666150630171433 26467407 PMC4761629

[B20] Boulle F. Van Den Hove D. L. Jakob S. B. Rutten B. P. Hamon M. Van Os J. (2012). Epigenetic regulation of the BDNF gene: Implications for psychiatric disorders. *Mol. Psychiatry* 17 584–596. 10.1038/mp.2011.107 21894152

[B21] Brennand K. J. Simone A. Jou J. Gelboin-Burkhart C. Tran N. Sangar S. (2011). Modelling schizophrenia using human induced pluripotent stem cells. *Nature* 473 221–225. 10.1038/nature09915 21490598 PMC3392969

[B22] Burnell E. S. Irvine M. Fang G. Sapkota K. Jane D. E. Monaghan D. T. (2019). Positive and Negative Allosteric Modulators of N-Methyl-d-aspartate (n.d.) Receptors: Structure-Activity Relationships and Mechanisms of Action. *J. Med. Chem.* 62 3–23. 10.1021/acs.jmedchem.7b01640 29446949 PMC6368479

[B23] Carrard A. Salzmann A. Malafosse A. Karege F. (2011). Increased DNA methylation status of the serotonin receptor 5HTR1A gene promoter in schizophrenia and bipolar disorder. *J. Affect. Disord.* 132 450–453. 10.1016/j.jad.2011.03.018 21453976

[B24] Chang P.-K. Chu J. Tsai Y.-T. Lai Y.-H. Chen J.-C. (2020). Dopamine D3 receptor and GSK3β signaling mediate deficits in novel object recognition memory within dopamine transporter knockdown mice. *J. Biomed. Sci.* 27:16. 10.1186/s12929-019-0613-y 31900153 PMC6942274

[B25] Chao H. T. Chen H. Samaco R. C. Xue M. Chahrour M. Yoo J. (2010). Dysfunction in GABA signalling mediates autism-like stereotypies and Rett syndrome phenotypes. *Nature* 468 263–269. 10.1038/nature09582 21068835 PMC3057962

[B26] Cheah S. Y. Lawford B. R. Young R. M. Morris C. P. Voisey J. (2017). mRNA Expression and DNA Methylation Analysis of Serotonin Receptor 2A (HTR2A) in the Human Schizophrenic Brain. *Genes* 8:14. 10.3390/genes8010014 28054990 PMC5295009

[B27] Chen C. M. Stanford A. D. Mao X. Abi-Dargham A. Shungu D. C. Lisanby S. H. (2014). GABA level, gamma oscillation, and working memory performance in schizophrenia. *Neuroimage Clin.* 4 531–539. 10.1016/j.nicl.2014.03.007 24749063 PMC3989525

[B28] Chen J. Zang Z. Braun U. Schwarz K. Harneit A. Kremer T. (2020). Association of a reproducible epigenetic risk profile for schizophrenia with brain methylation and function. *JAMA Psychiatry* 77 628–636. 10.1001/jamapsychiatry.2019.4792 32049268 PMC7042900

[B29] Chen R. Davis L. K. Guter S. Wei Q. Jacob S. Potter M. H. (2017). Leveraging blood serotonin as an endophenotype to identify de novo and rare variants involved in autism. *Mol. Autism.* 8:14. 10.1186/s13229-017-0130-3 28344757 PMC5361831

[B30] Chen Y. Sharma R. P. Costa R. H. Costa E. Grayson D. R. (2002). On the epigenetic regulation of the human reelin promoter. *Nucleic Acids Res.* 30 2930–2939. 10.1093/nar/gkf401 12087179 PMC117056

[B31] Cheng J. Wang Y. Zhou K. Wang L. Li J. Zhuang Q. (2014). Male-specific association between dopamine receptor D4 gene methylation and schizophrenia. *PLoS One* 9:e89128. 10.1371/journal.pone.0089128 24586542 PMC3929639

[B32] Cieślik P. Radulska A. Burnat G. Kalinowski L. Wieroñska J. M. (2021). Serotonergic-muscarinic interaction within the prefrontal cortex as a novel target to reverse schizophrenia-related cognitive symptoms. *Int. J. Mol. Sci.* 22:8612. 10.3390/ijms22168612 34445318 PMC8395335

[B33] GBD 2019 Diseases and Injuries Collaborators (2020). Global burden of 369 diseases and injuries in 204 countries and territories, 1990-2019: A systematic analysis for the global burden of disease study 2019. *Lancet* 396 1204–1222. 10.1016/s0140-6736(20)30925-9 33069326 PMC7567026

[B34] Schizophrenia Working Group of the Psychiatric Genomics Consortium (2014). Biological insights from 108 schizophrenia-associated genetic loci. *Nature* 511 421–427. 10.1038/nature13595 25056061 PMC4112379

[B35] Corlett P. R. Honey G. D. Krystal J. H. Fletcher P. C. (2011). Glutamatergic model psychoses: Prediction error, learning, and inference. *Neuropsychopharmacology* 36 294–315. 10.1038/npp.2010.163 20861831 PMC3055519

[B36] da Silveira Cruz-Machado S. Guissoni Campos L. M. Fadini C. C. Anderson G. Markus R. P. Pinato L. (2021). Disrupted nocturnal melatonin in autism: Association with tumor necrosis factor and sleep disturbances. *J. Pineal. Res.* 70:e12715. 10.1111/jpi.12715 33421193

[B37] Dai D. Cheng J. Zhou K. Lv Y. Zhuang Q. Zheng R. (2014). Significant association between DRD3 gene body methylation and schizophrenia. *Psychiatry Res.* 220 772–777. 10.1016/j.psychres.2014.08.032 25262640

[B38] Day J. J. Sweatt J. D. (2012). Epigenetic treatments for cognitive impairments. *Neuropsychopharmacology* 37 247–260. 10.1038/npp.2011.85 21593731 PMC3238093

[B39] de Bruin N. M. Kruse C. G. (2015). 5-HT6 receptor antagonists: Potential efficacy for the treatment of cognitive impairment in schizophrenia. *Curr. Pharm. Des.* 21 3739–3759. 10.2174/1381612821666150605112105 26044973

[B40] Desai A. Benner L. Wu R. Gertsik L. Maruff P. Light G. A. (2021). Phase 1 randomized study on the safety, tolerability, and pharmacodynamic cognitive and electrophysiological effects of a dopamine D(1) receptor positive allosteric modulator in patients with schizophrenia. *Neuropsychopharmacology* 46 1145–1151. 10.1038/s41386-020-00908-0 33203954 PMC8182805

[B41] Deutsch S. I. Luyo Z. N. M. Burket J. A. (2022). Targeted NMDA Receptor Interventions for Autism: Developmentally Determined Expression of GluN2B and GluN2A-Containing Receptors and Balanced Allosteric Modulatory Approaches. *Biomolecules* 12:181. 10.3390/biom12020181 35204682 PMC8961601

[B42] Dias V. V. Brissos S. Frey B. N. Andreazza A. C. Cardoso C. Kapczinski F. (2009). Cognitive function and serum levels of brain-derived neurotrophic factor in patients with bipolar disorder. *Bipolar Disord.* 11 663–671. 10.1111/j.1399-5618.2009.00733.x 19689509

[B43] Dickerson F. Stallings C. Origoni A. Vaughan C. Khushalani S. Yolken R. (2013). Elevated C-reactive protein and cognitive deficits in individuals with bipolar disorder. *J. Affect. Disord.* 150 456–459. 10.1016/j.jad.2013.04.039 23684514

[B44] Dong E. Agis-Balboa R. C. Simonini M. V. Grayson D. R. Costa E. Guidotti A. (2005). Reelin and glutamic acid decarboxylase67 promoter remodeling in an epigenetic methionine-induced mouse model of schizophrenia. *Proc. Natl. Acad. Sci. U.S.A.* 102 12578–12583. 10.1073/pnas.0505394102 16113080 PMC1194936

[B45] Ehlis A. C. Reif A. Herrmann M. J. Lesch K. P. Fallgatter A. J. (2007). Impact of catechol-O-methyltransferase on prefrontal brain functioning in schizophrenia spectrum disorders. *Neuropsychopharmacology* 32 162–170. 10.1038/sj.npp.1301151 16823382

[B46] Fatemi S. H. Earle J. A. Mcmenomy T. (2000). Reduction in Reelin immunoreactivity in hippocampus of subjects with schizophrenia, bipolar disorder and major depression. *Mol. Psychiatry* 5:571. 10.1038/sj.mp.4000783 11126396

[B47] Fatemi S. H. Reutiman T. J. Folsom T. D. Rooney R. J. Patel D. H. Thuras P. D. (2010). mRNA and protein levels for GABAAalpha4, alpha5, beta1 and GABABR1 receptors are altered in brains from subjects with autism. *J. Autism Dev. Disord.* 40 743–750. 10.1007/s10803-009-0924-z 20066485 PMC2865581

[B48] Fee C. Banasr M. Sibille E. (2017). Somatostatin-positive gamma-aminobutyric acid interneuron deficits in depression: Cortical microcircuit and therapeutic perspectives. *Biol. Psychiatry* 82 549–559. 10.1016/j.biopsych.2017.05.024 28697889 PMC5610074

[B49] Fillman S. G. Weickert T. W. Lenroot R. K. Catts S. V. Bruggemann J. M. Catts V. S. (2016). Elevated peripheral cytokines characterize a subgroup of people with schizophrenia displaying poor verbal fluency and reduced Broca’s area volume. *Mol. Psychiatry* 21 1090–1098. 10.1038/mp.2015.90 26194183 PMC4960447

[B50] First M. B. Gaebel W. Maj M. Stein D. J. Kogan C. S. Saunders J. B. (2021). An organization- and category-level comparison of diagnostic requirements for mental disorders in ICD-11 and DSM-5. *World Psychiatry* 20 34–51. 10.1002/wps.20825 33432742 PMC7801846

[B51] Fischer A. Sananbenesi F. Mungenast A. Tsai L. H. (2010). Targeting the correct HDAC(s) to treat cognitive disorders. *Trends Pharmacol. Sci.* 31 605–617. 10.1016/j.tips.2010.09.003 20980063

[B52] Fleischhacker W. W. Podhorna J. Gröschl M. Hake S. Zhao Y. Huang S. (2021). Efficacy and safety of the novel glycine transporter inhibitor BI 425809 once daily in patients with schizophrenia: A double-blind, randomised, placebo-controlled phase 2 study. *Lancet Psychiatry* 8 191–201. 10.1016/s2215-0366(20)30513-7 33610228

[B53] Frampton J. E. (2016). Vortioxetine: A Review in Cognitive Dysfunction in Depression. *Drugs* 76 1675–1682. 10.1007/s40265-016-0655-3 27807822

[B54] Fregeac J. Colleaux L. Nguyen L. S. (2016). The emerging roles of MicroRNAs in autism spectrum disorders. *Neurosci. Biobehav. Rev.* 71 729–738. 10.1016/j.neubiorev.2016.10.018 27793596

[B55] Fries G. R. Li Q. Mcalpin B. Rein T. Walss-Bass C. Soares J. C. (2016). The role of DNA methylation in the pathophysiology and treatment of bipolar disorder. *Neurosci. Biobehav. Rev.* 68 474–488. 10.1016/j.neubiorev.2016.06.010 27328785 PMC5003658

[B56] Frydecka D. Misiak B. Pawlak-Adamska E. Karabon L. Tomkiewicz A. Sedlaczek P. (2015). Interleukin-6: The missing element of the neurocognitive deterioration in schizophrenia? The focus on genetic underpinnings, cognitive impairment and clinical manifestation. *Eur. Arch. Psychiatry Clin. Neurosci.* 265 449–459. 10.1007/s00406-014-0533-5 25214388 PMC4540774

[B57] Fung L. K. Mahajan R. Nozzolillo A. Bernal P. Krasner A. Jo B. (2016). Pharmacologic Treatment of Severe Irritability and Problem Behaviors in Autism: A Systematic Review and Meta-analysis. *Pediatrics* 137:S124–S135. 10.1542/peds.2015-2851K 26908468

[B58] Gilbert T. M. Zürcher N. R. Wu C. J. Bhanot A. Hightower B. G. Kim M. (2019). PET neuroimaging reveals histone deacetylase dysregulation in schizophrenia. *J. Clin. Invest.* 129 364–372. 10.1172/jci123743 30530989 PMC6307962

[B59] Girdhar K. Hoffman G. E. Jiang Y. Brown L. Kundakovic M. Hauberg M. E. (2018). Cell-specific histone modification maps in the human frontal lobe link schizophrenia risk to the neuronal epigenome. *Nat. Neurosci.* 21 1126–1136. 10.1038/s41593-018-0187-0 30038276 PMC6063773

[B60] Glinert A. Turjeman S. Elliott E. Koren O. (2022). Microbes, metabolites and (synaptic) malleability, oh my! the effect of the microbiome on synaptic plasticity. *Biol. Rev. Camb. Philos. Soc.* 97 582–599. 10.1111/brv.12812 34734461 PMC9298272

[B61] Gobshtis N. Tfilin M. Fraifeld V. E. Turgeman G. (2021). Transplantation of mesenchymal stem cells causes long-term alleviation of schizophrenia-like behaviour coupled with increased neurogenesis. *Mol. Psychiatry* 26 4448–4463. 10.1038/s41380-019-0623-x 31827249

[B62] Grace A. A. (2016). Dysregulation of the dopamine system in the pathophysiology of schizophrenia and depression. *Nat. Rev. Neurosci.* 17 524–532. 10.1038/nrn.2016.57 27256556 PMC5166560

[B63] Grande I. Berk M. Birmaher B. Vieta E. (2016). Bipolar disorder. *Lancet* 387 1561–1572. 10.1016/s0140-6736(15)00241-x 26388529

[B64] Grayson D. R. Guidotti A. (2013). The dynamics of DNA methylation in schizophrenia and related psychiatric disorders. *Neuropsychopharmacology* 38 138–166. 10.1038/npp.2012.125 22948975 PMC3521968

[B65] Green E. K. Rees E. Walters J. T. Smith K. G. Forty L. Grozeva D. (2016). Copy number variation in bipolar disorder. *Mol. Psychiatry* 21 89–93. 10.1038/mp.2014.174 25560756 PMC5038134

[B66] Green M. J. Cairns M. J. Wu J. Dragovic M. Jablensky A. Tooney P. A. (2013). Genome-wide supported variant MIR137 and severe negative symptoms predict membership of an impaired cognitive subtype of schizophrenia. *Mol. Psychiatry* 18 774–780. 10.1038/mp.2012.84 22733126

[B67] Green M. J. Matheson S. L. Shepherd A. Weickert C. S. Carr V. J. (2011). Brain-derived neurotrophic factor levels in schizophrenia: A systematic review with meta-analysis. *Mol. Psychiatry* 16 960–972. 10.1038/mp.2010.88 20733577

[B68] Greenwood T. A. Lazzeroni L. C. Maihofer A. X. Swerdlow N. R. Calkins M. E. Freedman R. (2019). Genome-wide association of endophenotypes for schizophrenia from the Consortium on the Genetics of Schizophrenia (COGS) study. *JAMA Psychiatry* 76 1274–1284. 10.1001/jamapsychiatry.2019.2850 31596458 PMC6802253

[B69] Guan J. S. Haggarty S. J. Giacometti E. Dannenberg J. H. Joseph N. Gao J. (2009). HDAC2 negatively regulates memory formation and synaptic plasticity. *Nature* 459 55–60. 10.1038/nature07925 19424149 PMC3498958

[B70] Guan L. Y. Hou W. L. Zhu Z. H. Cao J. Q. Tang Z. Yin X. Y. (2021). Associations among gonadal hormone, triglycerides and cognitive decline in female patients with major depressive disorders. *J. Psychiatr. Res.* 143 580–586. 10.1016/j.jpsychires.2020.11.022 33213891

[B71] Guercio G. D. Thomas M. E. Cisneros-Franco J. M. Voss P. Panizzutti R. De Villers-Sidani E. (2019). Improving cognitive training for schizophrenia using neuroplasticity enhancers: Lessons from decades of basic and clinical research. *Schizophr. Res.* 207 80–92. 10.1016/j.schres.2018.04.028 29730045

[B72] Guidotti A. Auta J. Davis J. M. Di-Giorgi-Gerevini V. Dwivedi Y. Grayson D. R. (2000). Decrease in reelin and glutamic acid decarboxylase67 (GAD67) expression in schizophrenia and bipolar disorder: A postmortem brain study. *Arch. Gen. Psychiatry* 57 1061–1069. 10.1001/archpsyc.57.11.1061 11074872

[B73] Guo C. Liu Y. Fang M.-S. Li Y. Li W. Mahaman Y. A. R. (2020). ω-3PUFAs improve cognitive impairments through ser133 phosphorylation of CREB upregulating BDNF/TrkB signal in schizophrenia. *Neurotherapeutics* 17 1271–1286. 10.1007/s13311-020-00859-w 32367475 PMC7609637

[B74] Gupta S. Kim S. Y. Artis S. Molfese D. L. Schumacher A. Sweatt J. D. (2010). Histone methylation regulates memory formation. *J. Neurosci.* 30 3589–3599. 10.1523/jneurosci.3732-09.2010 20219993 PMC2859898

[B75] Haas S. S. Antonucci L. A. Wenzel J. Ruef A. Biagianti B. Paolini M. (2021). A multivariate neuromonitoring approach to neuroplasticity-based computerized cognitive training in recent onset psychosis. *Neuropsychopharmacology* 46 828–835. 10.1038/s41386-020-00877-4 33027802 PMC8027389

[B76] Hagi K. Nosaka T. Dickinson D. Lindenmayer J. P. Lee J. Friedman J. (2021). Association between cardiovascular risk factors and cognitive impairment in people with schizophrenia: A systematic review and meta-analysis. *JAMA psychiatry* 78 510–518. 10.1001/jamapsychiatry.2021.0015 33656533 PMC7931134

[B77] Harrell C. R. Volarevic A. Djonov V. Volarevic V. (2021). Mesenchymal stem cell-derived exosomes as new remedy for the treatment of neurocognitive disorders. *Int. J. Mol. Sci.* 22:1433. 10.3390/ijms22031433 33535376 PMC7867043

[B78] Hasan A. Mitchell A. Schneider A. Halene T. Akbarian S. (2013). Epigenetic dysregulation in schizophrenia: Molecular and clinical aspects of histone deacetylase inhibitors. *Eur. Arch. Psychiatry Clin. Neurosci.* 263 273–284. 10.1007/s00406-013-0395-2 23381549

[B79] Hashemi E. Ariza J. Rogers H. Noctor S. C. Martínez-Cerdeño V. (2017). The number of parvalbumin-expressing interneurons is decreased in the prefrontal cortex in autism. *Cereb. Cortex* 27 1931–1943. 10.1093/cercor/bhw021 26922658 PMC6074948

[B80] Hidalgo S. Campusano J. Hodge J. (2021). The drosophila ortholog of the schizophrenia-associated CACNA1A and CACNA1B voltage-gated calcium channels regulate memory, sleep and circadian rhythms. *Neurobiol. Dis.* 155:105394. 10.1016/j.nbd.2021.105394 34015490

[B81] Huang H. S. Matevossian A. Whittle C. Kim S. Y. Schumacher A. Baker S. P. (2007). Prefrontal dysfunction in schizophrenia involves mixed-lineage leukemia 1-regulated histone methylation at GABAergic gene promoters. *J. Neurosci.* 27 11254–11262. 10.1523/jneurosci.3272-07.2007 17942719 PMC6673022

[B82] Huang X. Fei G. Q. Liu W. J. Ding J. Wang Y. Wang H. (2020). Adipose-derived mesenchymal stem cells protect against CMS-induced depression-like behaviors in mice via regulating the Nrf2/HO-1 and TLR4/NF-κB signaling pathways. *Acta Pharmacol. Sin.* 41 612–619. 10.1038/s41401-019-0317-6 31796867 PMC7468309

[B83] Hubbard L. Rees E. Morris D. W. Lynham A. J. Richards A. L. Pardiñas A. F. (2021). Rare Copy Number Variants Are Associated With Poorer Cognition in Schizophrenia. *Biol. Psychiatry* 90 28–34. 10.1016/j.biopsych.2020.11.025 33678419

[B84] Hui L. Yin X. L. Chen J. Yin X. Y. Zhu H. L. Li J. (2019). Association between decreased HDL levels and cognitive deficits in patients with bipolar disorder: A pilot study. *Int. J. Bipolar Disord.* 7:25. 10.1186/s40345-019-0159-7 31761966 PMC6875532

[B85] Jaffe A. E. Gao Y. Deep-Soboslay A. Tao R. Hyde T. M. Weinberger D. R. (2016). Mapping DNA methylation across development, genotype and schizophrenia in the human frontal cortex. *Nat. Neurosci.* 19 40–47. 10.1038/nn.4181 26619358 PMC4783176

[B86] Jakovcevski M. Akbarian S. (2012). Epigenetic mechanisms in neurological disease. *Nat. Med.* 18 1194–1204. 10.1038/nm.2828 22869198 PMC3596876

[B87] Jakovcevski M. Bharadwaj R. Straubhaar J. Gao G. Gavin D. P. Jakovcevski I. (2013). Prefrontal cortical dysfunction after overexpression of histone deacetylase 1. *Biol. Psychiatry* 74 696–705. 10.1016/j.biopsych.2013.03.020 23664640 PMC3797203

[B88] Jakovcevski M. Ruan H. Shen E. Y. Dincer A. Javidfar B. Ma Q. (2015). Neuronal Kmt2a/Mll1 histone methyltransferase is essential for prefrontal synaptic plasticity and working memory. *J. Neurosci.* 35 5097–5108. 10.1523/JNEUROSCI.3004-14.2015 25834037 PMC4380991

[B89] Jauhar S. Johnstone M. Mckenna P. (2022). Schizophrenia. *Lancet* 399 473–486. 10.1016/s0140-6736(21)01730-x 35093231

[B90] Javidfar B. Park R. Kassim B. S. Bicks L. K. Akbarian S. (2017). The epigenomics of schizophrenia, in the mouse. *Am. J. Med. Genet. Part B Neuropsychiatr. Genet.* 174 631–640. 10.1002/ajmg.b.32566 28699694 PMC5573750

[B91] Javitt D. C. Carter C. S. Krystal J. H. Kantrowitz J. T. Girgis R. R. Kegeles L. S. (2018). Utility of Imaging-Based Biomarkers for Glutamate-Targeted Drug Development in Psychotic Disorders: A Randomized Clinical Trial. *JAMA Psychiatry* 75 11–19. 10.1001/jamapsychiatry.2017.3572 29167877 PMC5833531

[B92] Jin K. Lu J. Yu Z. Shen Z. Li H. Mou T. (2020). Linking peripheral IL-6, IL-1β and hypocretin-1 with cognitive impairment from major depression. *J. Affect. Disord.* 277 204–211. 10.1016/j.jad.2020.08.024 32829196

[B93] Jorm A. F. Patten S. B. Brugha T. S. Mojtabai R. (2017). Has increased provision of treatment reduced the prevalence of common mental disorders? Review of the evidence from four countries. *World Psychiatry* 16 90–99. 10.1002/wps.20388 28127925 PMC5269479

[B94] Joseph J. Depp C. Martin A. S. Daly R. E. Glorioso D. K. Palmer B. W. (2015). Associations of high sensitivity C-reactive protein levels in schizophrenia and comparison groups. *Schizophr. Res.* 168 456–460. 10.1016/j.schres.2015.08.019 26341579 PMC4591213

[B95] Kathuria A. Lopez-Lengowski K. Jagtap S. S. Mcphie D. Perlis R. H. Cohen B. M. (2020a). Transcriptomic landscape and functional characterization of induced pluripotent stem cell-derived cerebral organoids in schizophrenia. *JAMA Psychiatry* 77 745–754. 10.1001/jamapsychiatry.2020.0196 32186681 PMC7081156

[B96] Kathuria A. Lopez-Lengowski K. Vater M. Mcphie D. Cohen B. M. Karmacharya R. (2020b). Transcriptome analysis and functional characterization of cerebral organoids in bipolar disorder. *Genome Med.* 12:34. 10.1186/s13073-020-00733-6 32306996 PMC7168850

[B97] Keefe R. S. E. Harvey P. D. Khan A. Saoud J. B. Staner C. Davidson M. (2018). Cognitive Effects of MIN-101 in Patients With Schizophrenia and Negative Symptoms: Results From a Randomized Controlled Trial. *J. Clin. Psychiatry* 79:17m11753. 10.4088/JCP.17m11753 29873956

[B98] Keller S. Errico F. Zarrilli F. Florio E. Punzo D. Mansueto S. (2014). DNA methylation state of BDNF gene is not altered in prefrontal cortex and striatum of schizophrenia subjects. *Psychiatry Res.* 220 1147–1150. 10.1016/j.psychres.2014.08.022 25219617

[B99] Kendall K. M. Bracher-Smith M. Fitzpatrick H. Lynham A. Rees E. Escott-Price V. (2019a). Cognitive performance and functional outcomes of carriers of pathogenic copy number variants: Analysis of the UK biobank. *Br. J. Psychiatry* 214 297–304. 10.1192/bjp.2018.301 30767844 PMC6520248

[B100] Kendall K. M. Rees E. Bracher-Smith M. Legge S. Riglin L. Zammit S. (2019b). Association of rare copy number variants with risk of depression. *JAMA Psychiatry* 76 818–825. 10.1001/jamapsychiatry.2019.0566 30994872 PMC6583866

[B101] Kim H. Ährlund-Richter S. Wang X. Deisseroth K. Carlén M. (2016). Prefrontal parvalbumin neurons in control of attention. *Cell* 164 208–218. 10.1016/j.cell.2015.11.038 26771492 PMC4715187

[B102] King S. Holleran L. Mothersill D. Patlola S. Rokita K. Mcmanus R. (2021). Early life adversity, functional connectivity and cognitive performance in schizophrenia: The mediating role of IL-6. *Brain Behav. Immun.* 98 388–396. 10.1016/j.bbi.2021.06.016 34242739

[B103] Kirov G. Pocklington A. J. Holmans P. Ivanov D. Ikeda M. Ruderfer D. (2012). De novo CNV analysis implicates specific abnormalities of postsynaptic signalling complexes in the pathogenesis of schizophrenia. *Mol. Psychiatry* 17 142–153. 10.1038/mp.2011.154 22083728 PMC3603134

[B104] Klempan T. A. Sequeira A. Canetti L. Lalovic A. Ernst C. Ffrench-Mullen J. (2009). Altered expression of genes involved in ATP biosynthesis and GABAergic neurotransmission in the ventral prefrontal cortex of suicides with and without major depression. *Mol. Psychiatry* 14 175–189. 10.1038/sj.mp.4002110 17938633

[B105] Knöchel C. Kniep J. Cooper J. D. Stäblein M. Wenzler S. Sarlon J. (2017). Altered apolipoprotein C expression in association with cognition impairments and hippocampus volume in schizophrenia and bipolar disorder. *Eur. Arch. Psychiatry Clin. Neurosci.* 267 199–212. 10.1007/s00406-016-0724-3 27549216

[B106] Kocerha J. Dwivedi Y. Brennand K. J. (2015). Noncoding RNAs and neurobehavioral mechanisms in psychiatric disease. *Mol. Psychiatry* 20 677–684. 10.1038/mp.2015.30 25824307 PMC4440836

[B107] Kocerha J. Faghihi M. A. Lopez-Toledano M. A. Huang J. Ramsey A. J. Caron M. G. (2009). MicroRNA-219 modulates NMDA receptor-mediated neurobehavioral dysfunction. *Proc. Natl. Acad. Sci. U.S.A.* 106 3507–3512. 10.1073/pnas.0805854106 19196972 PMC2651305

[B108] Kogan S. Ospina L. H. Kimhy D. (2018). Inflammation in individuals with schizophrenia - implications for neurocognition and daily function. *Brain Behav. Immun.* 74 296–299. 10.1016/j.bbi.2018.09.016 30218782 PMC6805148

[B109] Kogan S. Ospina L. H. Mittal V. A. Kimhy D. (2020). The impact of inflammation on neurocognition and risk for psychosis: A critical review. *Eur. Arch. Psychiatry Clin. Neurosci.* 270 793–802. 10.1007/s00406-019-01073-2 31620871 PMC7160015

[B110] Kort N. S. Ford J. M. Roach B. J. Gunduz-Bruce H. Krystal J. H. Jaeger J. (2017). Role of N-methyl-D-aspartate receptors in action-based predictive coding deficits in schizophrenia. *Biol. Psychiatry* 81 514–524. 10.1016/j.biopsych.2016.06.019 27647218 PMC5203970

[B111] Kudo N. Yamamori H. Ishima T. Nemoto K. Yasuda Y. Fujimoto M. (2018). Plasma levels of soluble tumor necrosis factor receptor 2 (sTNFR2) are associated with hippocampal volume and cognitive performance in patients with schizophrenia. *Int. J. Neuropsychopharmacol.* 21 631–639. 10.1093/ijnp/pyy013 29529289 PMC6031046

[B112] Labouesse M. A. Dong E. Grayson D. R. Guidotti A. Meyer U. (2015). Maternal immune activation induces GAD1 and GAD2 promoter remodeling in the offspring prefrontal cortex. *Epigenetics* 10 1143–1155. 10.1080/15592294.2015.1114202 26575259 PMC4844233

[B113] Lai M. Lombardo M. Baron-Cohen S. (2014). Autism. *Lancet* 383 896–910. 10.1016/s0140-6736(13)61539-1 24074734

[B114] Lemonde S. Turecki G. Bakish D. Du L. Hrdina P. D. Bown C. D. (2003). Impaired repression at a 5-hydroxytryptamine 1A receptor gene polymorphism associated with major depression and suicide. *J. Neurosci.* 23 8788–8799. 10.1523/jneurosci.23-25-08788.2003 14507979 PMC6740417

[B115] Lencz T. Knowles E. Davies G. Guha S. Liewald D. C. Starr J. M. (2014). Molecular genetic evidence for overlap between general cognitive ability and risk for schizophrenia: A report from the cognitive genomics consorTium (COGENT). *Mol. Psychiatry* 19 168–174. 10.1038/mp.2013.166 24342994 PMC3968799

[B116] Lencz T. Malhotra A. K. (2015). Targeting the schizophrenia genome: A fast track strategy from GWAS to clinic. *Mol. Psychiatry* 20 820–826. 10.1038/mp.2015.28 25869805 PMC4486648

[B117] Levinson A. J. Fitzgerald P. B. Favalli G. Blumberger D. M. Daigle M. Daskalakis Z. J. (2010). Evidence of cortical inhibitory deficits in major depressive disorder. *Biol. Psychiatry* 67 458–464. 10.1016/j.biopsych.2009.09.025 19922906

[B118] Lewis D. A. (2014). Inhibitory neurons in human cortical circuits: Substrate for cognitive dysfunction in schizophrenia. *Curr. Opin. Neurobiol.* 26 22–26. 10.1016/j.conb.2013.11.003 24650500 PMC4024332

[B119] Lewis D. A. Cho R. Y. Carter C. S. Eklund K. Forster S. Kelly M. A. (2008). Subunit-selective modulation of GABA type A receptor neurotransmission and cognition in schizophrenia. *Am. J. Psychiatry* 165 1585–1593. 10.1176/appi.ajp.2008.08030395 18923067 PMC2876339

[B120] Lewis D. A. Curley A. A. Glausier J. R. Volk D. W. (2012). Cortical parvalbumin interneurons and cognitive dysfunction in schizophrenia. *Trends Neurosci.* 35 57–67. 10.1016/j.tins.2011.10.004 22154068 PMC3253230

[B121] Li C. T. Hsieh J. C. Wang S. J. Yang B. H. Bai Y. M. Lin W. C. (2012). Differential relations between fronto-limbic metabolism and executive function in patients with remitted bipolar I and bipolar II disorder. *Bipolar Disord.* 14 831–842. 10.1111/bdi.12017 23167933

[B122] Li Y. K. Hui C. L. Lee E. H. Chang W. C. Chan S. K. Leung C. M. (2014). Coupling physical exercise with dietary glucose supplement for treating cognitive impairment in schizophrenia: A theoretical model and future directions. *Early Interv. Psychiatry* 8 209–220. 10.1111/eip.12109 24224943

[B123] Li Z. Lai J. Zhang P. Ding J. Jiang J. Liu C. (2022). Multi-omics analyses of serum metabolome, gut microbiome and brain function reveal dysregulated microbiota-gut-brain axis in bipolar depression. *Mol. Psychiatry* [Epub ahead of print]. 10.1038/s41380-022-01569-9 35444255

[B124] Li Z. Wang G. Zhong S. Liao X. Lai S. Shan Y. (2020). Alleviation of cognitive deficits and high copper levels by an NMDA receptor antagonist in a rat depression model. *Compr. Psychiatry* 102:152200. 10.1016/j.comppsych.2020.152200 32892027

[B125] Lindenmayer J. P. Nasrallah H. Pucci M. James S. Citrome L. (2013). A systematic review of psychostimulant treatment of negative symptoms of schizophrenia: Challenges and therapeutic opportunities. *Schizophr. Res.* 147 241–252. 10.1016/j.schres.2013.03.019 23619055

[B126] Lintas C. Sacco R. Persico A. M. (2016). Differential methylation at the RELN gene promoter in temporal cortex from autistic and typically developing post-puberal subjects. *J. Neurodev. Disord.* 8:18. 10.1186/s11689-016-9151-z 27134686 PMC4850686

[B127] Liu J. Wang J. Xiao X. Lai X. Dai D. Zhang X. (2020). A hybrid correcting method considering heterozygous variations by a comprehensive probabilistic model. *BMC Genom.* 21:753. 10.1186/s12864-020-07008-9 33208104 PMC7677778

[B128] Liu L. Lv X. Zhou S. Liu Q. Wang J. Tian H. (2021). The effect of selective serotonin reuptake inhibitors on cognitive impairment in patients with depression: A prospective, multicenter, observational study. *J. Psychiatr. Res.* 141 26–33. 10.1016/j.jpsychires.2021.06.020 34171760

[B129] Liu P. Gao M. Liu Z. Zhang Y. Tu H. Lei L. (2021). Gut microbiome composition linked to inflammatory factors and cognitive functions in first-episode, drug-naive major depressive disorder patients. *Front. Neurosci.* 15:800764. 10.3389/fnins.2021.800764 35153660 PMC8831735

[B130] Liu Q. Chen M. X. Sun L. Wallis C. U. Zhou J. S. Ao L. J. (2019). Rational use of mesenchymal stem cells in the treatment of autism spectrum disorders. *World J. Stem Cells* 11 55–72. 10.4252/wjsc.v11.i2.55 30842805 PMC6397804

[B131] Liu S. Zhou L. Yuan H. Vieira M. Sanz-Clemente A. Badger J. D. II (2017). A rare variant identified within the GluN2B C-terminus in a patient with autism affects NMDA receptor surface expression and spine density. *J. Neurosci.* 37 4093–4102. 10.1523/jneurosci.0827-16.2017 28283559 PMC5391683

[B132] Lizano P. Lutz O. Ling G. Lee A. M. Eum S. Bishop J. R. (2019). Association of choroid plexus enlargement with cognitive, inflammatory, and structural phenotypes across the psychosis spectrum. *Am. J. Psychiatry* 176 564–572. 10.1176/appi.ajp.2019.18070825 31164007 PMC6676480

[B133] Locke T. M. Soden M. E. Miller S. M. Hunker A. Knakal C. Licholai J. A. (2018). Dopamine D(1) receptor-positive neurons in the lateral nucleus of the cerebellum contribute to cognitive behavior. *Biol. Psychiatry* 84 401–412. 10.1016/j.biopsych.2018.01.019 29478701 PMC6072628

[B134] Lv Y. T. Zhang Y. Liu M. Qiuwaxi J. N. Ashwood P. Cho S. C. (2013). Transplantation of human cord blood mononuclear cells and umbilical cord-derived mesenchymal stem cells in autism. *J. Transl. Med.* 11:196. 10.1186/1479-5876-11-196 23978163 PMC3765833

[B135] Lynham A. J. Jones I. R. Walters J. T. R. (2022). Web-based cognitive testing in psychiatric research: Validation and usability study. *J. Med. Internet Res.* 24:e28233. 10.2196/28233 35142640 PMC8874806

[B136] Marchetto M. C. Carromeu C. Acab A. Yu D. Yeo G. W. Mu Y. (2010). A model for neural development and treatment of Rett syndrome using human induced pluripotent stem cells. *Cell* 143 527–539. 10.1016/j.cell.2010.10.016 21074045 PMC3003590

[B137] Mealer R. G. Williams S. E. Daly M. J. Scolnick E. M. Cummings R. D. Smoller J. W. (2020). Glycobiology and schizophrenia: A biological hypothesis emerging from genomic research. *Mol. Psychiatry* 25 3129–3139. 10.1038/s41380-020-0753-1 32377000 PMC8081046

[B138] Meltzer H. Y. Li Z. Kaneda Y. Ichikawa J. (2003). Serotonin receptors: Their key role in drugs to treat schizophrenia. *Prog. Neuropsychopharmacol. Biol. Psychiatry* 27 1159–1172. 10.1016/j.pnpbp.2003.09.010 14642974

[B139] Meneses A. (1999). 5-HT system and cognition. *Neurosci. Biobehav. Rev.* 23 1111–1125. 10.1016/s0149-7634(99)00067-6 10643820

[B140] Miller B. H. Zeier Z. Xi L. Lanz T. A. Deng S. Strathmann J. (2012). MicroRNA-132 dysregulation in schizophrenia has implications for both neurodevelopment and adult brain function. *Proc. Natl. Acad. Sci. U.S.A.* 109 3125–3130. 10.1073/pnas.1113793109 22315408 PMC3286960

[B141] Miller B. J. Buckley P. Seabolt W. Mellor A. Kirkpatrick B. (2011). Meta-analysis of cytokine alterations in schizophrenia: Clinical status and antipsychotic effects. *Biol. Psychiatry* 70 663–671. 10.1016/j.biopsych.2011.04.013 21641581 PMC4071300

[B142] Millett C. E. Harder J. Locascio J. J. Shanahan M. Santone G. Fichorova R. N. (2020). TNF-α and its soluble receptors mediate the relationship between prior severe mood episodes and cognitive dysfunction in euthymic bipolar disorder. *Brain Behav. Immun.* 88 403–410. 10.1016/j.bbi.2020.04.003 32272224 PMC8577222

[B143] Millett C. E. Perez-Rodriguez M. Shanahan M. Larsen E. Yamamoto H. S. Bukowski C. (2021). C-reactive protein is associated with cognitive performance in a large cohort of euthymic patients with bipolar disorder. *Mol. Psychiatry* 26 4096–4105. 10.1038/s41380-019-0591-1 31740754

[B144] Misiak B. Stańczykiewicz B. Kotowicz K. Rybakowski J. K. Samochowiec J. Frydecka D. (2018). Cytokines and C-reactive protein alterations with respect to cognitive impairment in schizophrenia and bipolar disorder: A systematic review. *Schizophr. Res.* 192 16–29. 10.1016/j.schres.2017.04.015 28416092

[B145] Mitchell A. C. Jiang Y. Peter C. Akbarian S. (2015). Transcriptional regulation of GAD1 GABA synthesis gene in the prefrontal cortex of subjects with schizophrenia. *Schizophr. Res.* 167 28–34. 10.1016/j.schres.2014.10.020 25458568 PMC4417100

[B146] Mitelman S. A. Bralet M. C. Mehmet Haznedar M. Hollander E. Shihabuddin L. Hazlett E. A. (2018). Positron emission tomography assessment of cerebral glucose metabolic rates in autism spectrum disorder and schizophrenia. *Brain Imaging Behav.* 12 532–546. 10.1007/s11682-017-9721-z 28425060 PMC5648637

[B147] Moen M. J. Adams H. H. Brandsma J. H. Dekkers D. H. Akinci U. Karkampouna S. (2017). An interaction network of mental disorder proteins in neural stem cells. *Transl. Psychiatry* 7:e1082. 10.1038/tp.2017.52 28375211 PMC5416693

[B148] Moghaddam B. Javitt D. (2012). From revolution to evolution: The glutamate hypothesis of schizophrenia and its implication for treatment. *Neuropsychopharmacology* 37 4–15. 10.1038/npp.2011.181 21956446 PMC3238069

[B149] Mor M. Nardone S. Sams D. S. Elliott E. (2015). Hypomethylation of miR-142 promoter and upregulation of microRNAs that target the oxytocin receptor gene in the autism prefrontal cortex. *Mol. Autism* 6:46. 10.1186/s13229-015-0040-1 26273428 PMC4535255

[B150] Morrens M. Overloop C. Coppens V. Loots E. Van Den Noortgate M. Vandenameele S. (2022). The relationship between immune and cognitive dysfunction in mood and psychotic disorder: A systematic review and a meta-analysis. *Mol. Psychiatry* [Epub ahead of print]. 10.1038/s41380-022-01582-y 35484245 PMC9708549

[B151] Mould A. Hall N. Milosevic I. Tunbridge E. (2021). Targeting synaptic plasticity in schizophrenia: Insights from genomic studies. *Trends Mol. Med.* 27 1022–1032. 10.1016/j.molmed.2021.07.014 34419330

[B152] Nguyen L. S. Lepleux M. Makhlouf M. Martin C. Fregeac J. Siquier-Pernet K. (2016). Profiling olfactory stem cells from living patients identifies miRNAs relevant for autism pathophysiology. *Mol. Autism* 7:1. 10.1186/s13229-015-0064-6 26753090 PMC4705753

[B153] Nie J. Fang Y. Chen Y. Aidina A. Qiu Q. Zhao L. (2021). Characteristics of dysregulated proinflammatory cytokines and cognitive dysfunction in late-life depression and amnestic mild cognitive impairment. *Front. Immunol.* 12:803633. 10.3389/fimmu.2021.803633 35069588 PMC8767092

[B154] Northoff G. Sibille E. (2014). Why are cortical GABA neurons relevant to internal focus in depression? A cross-level model linking cellular, biochemical and neural network findings. *Mol. Psychiatry* 19 966–977. 10.1038/mp.2014.68 25048001 PMC4169738

[B155] Nuechterlein K. H. Green M. F. Kern R. S. Baade L. E. Barch D. M. Cohen J. D. (2008). The MATRICS consensus cognitive battery, part 1: Test selection, reliability, and validity. *Am. J. Psychiatry* 165 203–213. 10.1176/appi.ajp.2007.07010042 18172019

[B156] Ohi K. Hashimoto R. Ikeda M. Yamamori H. Yasuda Y. Fujimoto M. (2015). Glutamate networks implicate cognitive impairments in schizophrenia: Genome-wide association studies of 52 cognitive phenotypes. *Schizophr. Bull.* 41 909–918. 10.1093/schbul/sbu171 25537281 PMC4466179

[B157] O’Shea K. S. McInnis M. G. (2016). Neurodevelopmental origins of bipolar disorder: IPSC models. *Mol. Cell. Neurosci.* 73 63–83. 10.1016/j.mcn.2015.11.006 26608002

[B158] Pan S. Feng W. Li Y. Huang J. Chen S. Cui Y. (2021). The microRNA-195 - BDNF pathway and cognitive deficits in schizophrenia patients with minimal antipsychotic medication exposure. *Transl. Psychiatry* 11:117. 10.1038/s41398-021-01240-x 33558459 PMC7870897

[B159] Park H. Poo M. M. (2013). Neurotrophin regulation of neural circuit development and function. *Nat. Rev. Neurosci.* 14 7–23. 10.1038/nrn3379 23254191

[B160] Park K. M. Bowers W. J. (2010). Tumor necrosis factor-alpha mediated signaling in neuronal homeostasis and dysfunction. *Cell. Signal.* 22 977–983. 10.1016/j.cellsig.2010.01.010 20096353 PMC2860549

[B161] Paul S. M. Doherty J. J. Robichaud A. J. Belfort G. M. Chow B. Y. Hammond R. S. (2013). The major brain cholesterol metabolite 24(S)-hydroxycholesterol is a potent allosteric modulator of N-methyl-D-aspartate receptors. *J. Neurosci.* 33 17290–17300. 10.1523/jneurosci.2619-13.2013 24174662 PMC3812502

[B162] Perets N. Hertz S. London M. Offen D. (2018). Intranasal administration of exosomes derived from mesenchymal stem cells ameliorates autistic-like behaviors of BTBR mice. *Mol. Autism* 9:57. 10.1186/s13229-018-0240-6 30479733 PMC6249852

[B163] Perets N. Segal-Gavish H. Gothelf Y. Barzilay R. Barhum Y. Abramov N. (2017). Long term beneficial effect of neurotrophic factors-secreting mesenchymal stem cells transplantation in the BTBR mouse model of autism. *Behav. Brain Res.* 331 254–260. 10.1016/j.bbr.2017.03.047 28392323

[B164] Peter C. J. Akbarian S. (2011). Balancing histone methylation activities in psychiatric disorders. *Trends Mol. Med.* 17 372–379. 10.1016/j.molmed.2011.02.003 21429800 PMC3134618

[B165] Pizzo L. Jensen M. Polyak A. Rosenfeld J. A. Mannik K. Krishnan A. (2019). Rare variants in the genetic background modulate cognitive and developmental phenotypes in individuals carrying disease-associated variants. *Genet. Med.* 21 816–825. 10.1038/s41436-018-0266-3 30190612 PMC6405313

[B166] Poletti S. Mazza M. G. Calesella F. Vai B. Lorenzi C. Manfredi E. (2021). Circulating inflammatory markers impact cognitive functions in bipolar depression. *J. Psychiatr. Res.* 140 110–116. 10.1016/j.jpsychires.2021.05.071 34107379

[B167] Qin L. Ma K. Wang Z. J. Hu Z. Matas E. Wei J. (2018). Social deficits in Shank3-deficient mouse models of autism are rescued by histone deacetylase (HDAC) inhibition. *Nat. Neurosci.* 21 564–575. 10.1038/s41593-018-0110-8 29531362 PMC5876144

[B168] Qin X. Y. Wu H. T. Cao C. Loh Y. P. Cheng Y. (2017). A meta-analysis of peripheral blood nerve growth factor levels in patients with schizophrenia. *Mol. Psychiatry* 22 1306–1312. 10.1038/mp.2016.235 28070123

[B169] Qiu Y. Li S. Teng Z. Tan Y. Xu X. Yang M. (2022). Association between abnormal glycolipid level and cognitive dysfunction in drug-naïve patients with bipolar disorder. *J. Affect. Disord.* 297 477–485. 10.1016/j.jad.2021.10.100 34715186

[B170] Rao S. Martínez-Cengotitabengoa M. Yao Y. Guo Z. Xu Q. Li S. (2017). Peripheral blood nerve growth factor levels in major psychiatric disorders. *J. Psychiatr. Res.* 86 39–45. 10.1016/j.jpsychires.2016.11.012 27898323

[B171] Recio-Barbero M. Segarra R. Zabala A. González-Fraile E. González-Pinto A. Ballesteros J. (2021). Cognitive Enhancers in Schizophrenia: A Systematic Review and Meta-Analysis of Alpha-7 Nicotinic Acetylcholine Receptor Agonists for Cognitive Deficits and Negative Symptoms. *Front. Psychiatry* 12:631589. 10.3389/fpsyt.2021.631589 33889097 PMC8055861

[B172] Rees E. Kirov G. (2021). Copy number variation and neuropsychiatric illness. *Curr. Opin. Genet. Dev.* 68 57–63. 10.1016/j.gde.2021.02.014 33752146 PMC8219524

[B173] Rees E. Owen M. J. (2020). Translating insights from neuropsychiatric genetics and genomics for precision psychiatry. *Genome Med.* 12:43. 10.1186/s13073-020-00734-5 32349784 PMC7189552

[B174] Reichenberg A. Harvey P. D. Bowie C. R. Mojtabai R. Rabinowitz J. Heaton R. K. (2009). Neuropsychological function and dysfunction in schizophrenia and psychotic affective disorders. *Schizophr. Bull.* 35 1022–1029. 10.1093/schbul/sbn044 18495643 PMC2728814

[B175] Rogers G. B. Keating D. J. Young R. L. Wong M. L. Licinio J. Wesselingh S. (2016). From gut dysbiosis to altered brain function and mental illness: Mechanisms and pathways. *Mol. Psychiatry* 21 738–748. 10.1038/mp.2016.50 27090305 PMC4879184

[B176] Rong H. Liu T. B. Yang K. J. Yang H. C. Wu D. H. Liao C. P. (2011). MicroRNA-134 plasma levels before and after treatment for bipolar mania. *J. Psychiatr. Res.* 45 92–95. 10.1016/j.jpsychires.2010.04.028 20546789

[B177] Rudolph U. Möhler H. (2014). GABAA receptor subtypes: Therapeutic potential in Down syndrome, affective disorders, schizophrenia, and autism. *Annu. Rev. Pharmacol. Toxicol.* 54 483–507. 10.1146/annurev-pharmtox-011613-135947 24160694 PMC3997216

[B178] Salvi V. Di Salvo G. Korčáková J. Torriero S. Aragno E. Kolenič M. (2020). Insulin resistance is associated with verbal memory impairment in bipolar disorders. *J. Affect. Disord.* 266 610–614. 10.1016/j.jad.2020.01.145 32056934

[B179] Sánchez-Ortí J. V. Balanzá-Martínez V. Correa-Ghisays P. Selva-Vera G. Vila-Francés J. Magdalena-Benedito R. (2022). Specific metabolic syndrome components predict cognition and social functioning in people with type 2 diabetes mellitus and severe mental disorders. *Acta Psychiatr. Scand.* 146 215–226. 10.1111/acps.13433 35359023

[B180] Sanders S. J. He X. Willsey A. J. Ercan-Sencicek A. G. Samocha K. E. Cicek A. E. (2015). Insights into autism spectrum disorder genomic architecture and biology from 71 risk loci. *Neuron* 87 1215–1233. 10.1016/j.neuron.2015.09.016 26402605 PMC4624267

[B181] Santos B. González-Fraile E. Zabala A. Guillén V. Rueda J. R. Ballesteros J. (2018). Cognitive improvement of acetylcholinesterase inhibitors in schizophrenia. *J. Psychopharmacol.* 32 1155–1166. 10.1177/0269881118805496 30324844

[B182] Sarter M. Lustig C. Taylor S. F. (2012). Cholinergic contributions to the cognitive symptoms of schizophrenia and the viability of cholinergic treatments. *Neuropharmacology* 62 1544–1553. 10.1016/j.neuropharm.2010.12.001 21156184 PMC3920544

[B183] Satterstrom F. K. Kosmicki J. A. Wang J. Breen M. S. De Rubeis S. An J.-Y. (2020). Large-scale exome sequencing study implicates both developmental and functional changes in the neurobiology of autism. *Cell* 180 568–584.e23. 10.1016/j.cell.2019.12.036 31981491 PMC7250485

[B184] Scheggia D. Mastrogiacomo R. Mereu M. Sannino S. Straub R. E. Armando M. (2018). Publisher correction: Variations in dysbindin-1 are associated with cognitive response to antipsychotic drug treatment. *Nat. Commun.* 9:3560. 10.1038/s41467-018-06062-y 30158661 PMC6115376

[B185] Schroeder F. A. Gilbert T. M. Feng N. Taillon B. D. Volkow N. D. Innis R. B. (2017). Expression of HDAC2 but not HDAC1 transcript is reduced in dorsolateral prefrontal cortex of patients with schizophrenia. *ACS Chem. Neurosci.* 8 662–668. 10.1021/acschemneuro.6b00372 27959513 PMC5436730

[B186] Sedlazeck F. J. Rescheneder P. Smolka M. Fang H. Nattestad M. Von Haeseler A. (2018). Accurate detection of complex structural variations using single-molecule sequencing. *Nat. Methods* 15 461–468. 10.1038/s41592-018-0001-7 29713083 PMC5990442

[B187] Segal-Gavish H. Karvat G. Barak N. Barzilay R. Ganz J. Edry L. (2016). Mesenchymal stem cell transplantation promotes neurogenesis and ameliorates autism related behaviors in BTBR mice. *Autism Res.* 9 17–32. 10.1002/aur.1530 26257137

[B188] Selimbeyoglu A. Kim C. K. Inoue M. Lee S. Y. Hong A. S. O. Kauvar I. (2017). Modulation of prefrontal cortex excitation/inhibition balance rescues social behavior in CNTNAP2-deficient mice. *Sci. Transl. Med.* 9:eaah6733. 10.1126/scitranslmed.aah6733 28768803 PMC5723386

[B189] Selvaraj S. Arnone D. Cappai A. Howes O. (2014). Alterations in the serotonin system in schizophrenia: A systematic review and meta-analysis of postmortem and molecular imaging studies. *Neurosci. Biobehav. Rev.* 45 233–245. 10.1016/j.neubiorev.2014.06.005 24971825

[B190] Shao T. N. Yin G. Z. Yin X. L. Wu J. Q. Du X. D. Zhu H. L. (2017). Elevated triglyceride levels are associated with cognitive impairments among patients with major depressive disorder. *Compr. Psychiatry* 75 103–109. 10.1016/j.comppsych.2017.03.007 28342378

[B191] Sharma A. Gokulchandran N. Sane H. Nagrajan A. Paranjape A. Kulkarni P. (2013). Autologous bone marrow mononuclear cell therapy for autism: An open label proof of concept study. *Stem Cells Int.* 2013:623875. 10.1155/2013/623875 24062774 PMC3767048

[B192] Sharma R. P. Grayson D. R. Gavin D. P. (2008). Histone deactylase 1 expression is increased in the prefrontal cortex of schizophrenia subjects: Analysis of the national brain databank microarray collection. *Schizophr. Res.* 98 111–117. 10.1016/j.schres.2007.09.020 17961987 PMC2254186

[B193] Sharma S. Gonda X. Tarazi F. (2018). Autism spectrum disorder: Classification, diagnosis and therapy. *Pharmacol. Ther.* 190 91–104. 10.1016/j.pharmthera.2018.05.007 29763648

[B194] Sharon G. Cruz N. J. Kang D. W. Gandal M. J. Wang B. Kim Y. M. (2019). Human gut microbiota from autism spectrum disorder promote behavioral symptoms in mice. *Cell* 177 1600–1618.e17. 10.1016/j.cell.2019.05.004 31150625 PMC6993574

[B195] Shen E. Shulha H. Weng Z. Akbarian S. (2014). Regulation of histone H3K4 methylation in brain development and disease. *Philos. Trans. R. Soc. Lond. Series B Biol. Sci.* 369:20130514. 10.1098/rstb.2013.0514 25135975 PMC4142035

[B196] Smeland O. Bahrami S. Frei O. Shadrin A. O’connell K. Savage J. (2020). Genome-wide analysis reveals extensive genetic overlap between schizophrenia, bipolar disorder, and intelligence. *Mol. Psychiatry* 25 844–853. 10.1038/s41380-018-0332-x 30610197 PMC6609490

[B197] Smeland O. B. Frei O. Kauppi K. Hill W. D. Li W. Wang Y. (2017). Identification of genetic loci jointly influencing schizophrenia risk and the cognitive traits of verbal-numerical reasoning, reaction time, and general cognitive function. *JAMA Psychiatry* 74 1065–1075. 10.1001/jamapsychiatry.2017.1986 28746715 PMC5710474

[B198] Smith M. A. Ersavas T. Ferguson J. M. Liu H. Lucas M. C. Begik O. (2020). Molecular barcoding of native RNAs using nanopore sequencing and deep learning. *Genome Res.* 30 1345–1353. 10.1101/gr.260836.120 32907883 PMC7545146

[B199] Snyder M. A. Gao W. J. (2020). NMDA receptor hypofunction for schizophrenia revisited: Perspectives from epigenetic mechanisms. *Schizophr. Res.* 217 60–70. 10.1016/j.schres.2019.03.010 30979669 PMC7258307

[B200] Sofroniew M. V. Howe C. L. Mobley W. C. (2001). Nerve growth factor signaling, neuroprotection, and neural repair. *Annu. Rev. Neurosci.* 24 1217–1281. 10.1146/annurev.neuro.24.1.1217 11520933

[B201] Soliman M. A. Aboharb F. Zeltner N. Studer L. (2017). Pluripotent stem cells in neuropsychiatric disorders. *Mol. Psychiatry* 22 1241–1249. 10.1038/mp.2017.40 28322279 PMC5582162

[B202] Srivastava A. Dada O. Qian J. Al-Chalabi N. Fatemi A. B. Gerretsen P. (2021). Epigenetics of schizophrenia. *Psychiatry Res.* 305:114218. 10.1016/j.psychres.2021.114218 34638051

[B203] Stefansson H. Meyer-Lindenberg A. Steinberg S. Magnusdottir B. Morgen K. Arnarsdottir S. (2014). CNVs conferring risk of autism or schizophrenia affect cognition in controls. *Nature* 505 361–366. 10.1038/nature12818 24352232

[B204] Stellwagen D. Malenka R. C. (2006). Synaptic scaling mediated by glial TNF-alpha. *Nature* 440 1054–1059. 10.1038/nature04671 16547515

[B205] Sullivan P. F. Geschwind D. H. (2019). Defining the genetic, genomic, cellular, and diagnostic architectures of psychiatric disorders. *Cell* 177 162–183. 10.1016/j.cell.2019.01.015 30901538 PMC6432948

[B206] Sumiyoshi T. Stockmeier C. A. Overholser J. C. Dilley G. E. Meltzer H. Y. (1996). Serotonin1A receptors are increased in postmortem prefrontal cortex in schizophrenia. *Brain Res.* 708 209–214. 10.1016/0006-8993(95)01361-x 8720882

[B207] Sun Y. Baptista L. C. Roberts L. M. Jumbo-Lucioni P. Mcmahon L. L. Buford T. W. (2020). The gut microbiome as a therapeutic target for cognitive impairment. *J. Gerontol. A Biol. Sci. Med. Sci.* 75 1242–1250. 10.1093/gerona/glz281 31811292 PMC7302188

[B208] Tan T. Wang W. Williams J. Ma K. Cao Q. Yan Z. (2019). Stress exposure in dopamine D4 receptor knockout mice induces schizophrenia-like behaviors via disruption of GABAergic transmission. *Schizophr. Bull.* 45 1012–1023. 10.1093/schbul/sby163 30476265 PMC6737476

[B209] Tang C. Z. Yang J. T. Liu Q. H. Wang Y. R. Wang W. S. (2019). Up-regulated miR-192-5p expression rescues cognitive impairment and restores neural function in mice with depression via the Fbln2-mediated TGF-β1 signaling pathway. *FASEB J.* 33 606–618. 10.1096/fj.201800210RR 30118321

[B210] Tang W. Wang Y. Xu F. Fan W. Zhang Y. Fan K. (2020). Omega-3 fatty acids ameliorate cognitive dysfunction in schizophrenia patients with metabolic syndrome. *Brain Behav. Immun.* 88 529–534. 10.1016/j.bbi.2020.04.034 32304881

[B211] Teng Z. Wang L. Li S. Tan Y. Qiu Y. Wu C. (2021). Low BDNF levels in serum are associated with cognitive impairments in medication-naïve patients with current depressive episode in BD II and MDD. *J. Affect. Disord.* 293 90–96. 10.1016/j.jad.2021.06.018 34175594

[B212] Tessier C. Sweers K. Frajerman A. Bergaoui H. Ferreri F. Delva C. (2016). Membrane lipidomics in schizophrenia patients: A correlational study with clinical and cognitive manifestations. *Transl. Psychiatry* 6:e906. 10.1038/tp.2016.142 27701405 PMC5315538

[B213] Thalamuthu A. Mills N. Berger K. Minnerup H. Grotegerd D. Dannlowski U. (2022). Genome-wide interaction study with major depression identifies novel variants associated with cognitive function. *Mol. Psychiatry* 27 1111–1119. 10.1038/s41380-021-01379-5 34782712 PMC7612684

[B214] Thygesen J. H. Presman A. Harju-Seppänen J. Irizar H. Jones R. Kuchenbaecker K. (2021). Genetic copy number variants, cognition and psychosis: A meta-analysis and a family study. *Mol. Psychiatry* 26 5307–5319. 10.1038/s41380-020-0820-7 32719466 PMC8589646

[B215] Tremblay M. W. Jiang Y. H. (2019). DNA methylation and susceptibility to autism spectrum disorder. *Annu. Rev. Med.* 70 151–166. 10.1146/annurev-med-120417-091431 30691368 PMC6597259

[B216] Tseng C. J. Gilbert T. M. Catanese M. C. Hightower B. G. Peters A. T. Parmar A. J. (2020). *In vivo* human brain expression of histone deacetylases in bipolar disorder. *Transl. Psychiatry* 10:224. 10.1038/s41398-020-00911-5 32641695 PMC7343804

[B217] Tsivion-Visbord H. Perets N. Sofer T. Bikovski L. Goldshmit Y. Ruban A. (2020). Mesenchymal stem cells derived extracellular vesicles improve behavioral and biochemical deficits in a phencyclidine model of schizophrenia. *Transl. Psychiatry* 10:305. 10.1038/s41398-020-00988-y 32873780 PMC7463024

[B218] Uhlhaas P. J. Singer W. (2010). Abnormal neural oscillations and synchrony in schizophrenia. *Nat. Rev. Neurosci.* 11 100–113. 10.1038/nrn2774 20087360

[B219] Vadodaria K. C. Mertens J. Paquola A. Bardy C. Li X. Jappelli R. (2016). Generation of functional human serotonergic neurons from fibroblasts. *Mol. Psychiatry* 21 49–61. 10.1038/mp.2015.161 26503761

[B220] van Dijk E. L. Jaszczyszyn Y. Naquin D. Thermes C. (2018). The third revolution in sequencing technology. *Trends Genet.* 34 666–681. 10.1016/j.tig.2018.05.008 29941292

[B221] Van Rheenen T. E. Mcintyre R. S. Balanzá-Martínez V. Berk M. Rossell S. L. (2021). Cumulative cardiovascular disease risk and triglycerides differentially relate to subdomains of executive function in bipolar disorder; preliminary findings. *J. Affect. Disord.* 278 556–562. 10.1016/j.jad.2020.09.104 33022441

[B222] Van Rheenen T. E. Rossell S. L. (2014). An empirical evaluation of the MATRICS consensus cognitive battery in bipolar disorder. *Bipolar Disord.* 16 318–325. 10.1111/bdi.12134 24119238

[B223] Veldic M. Kadriu B. Maloku E. Agis-Balboa R. C. Guidotti A. Davis J. M. (2007). Epigenetic mechanisms expressed in basal ganglia GABAergic neurons differentiate schizophrenia from bipolar disorder. *Schizophr. Res.* 91 51–61. 10.1016/j.schres.2006.11.029 17270400 PMC1876737

[B224] Veselinović T. Neuner I. (2022). Progress and pitfalls in developing agents to treat neurocognitive deficits associated with schizophrenia. *CNS Drugs* 36 819–858. 10.1007/s40263-022-00935-z 35831706 PMC9345797

[B225] Vieta E. Berk M. Schulze T. Carvalho A. Suppes T. Calabrese J. (2018). Bipolar disorders. *Nat. Rev. Dis. Primers* 4:18008. 10.1038/nrdp.2018.8 29516993

[B226] Viola T. W. Fries G. R. (2019). A promising era for epigenetic research: Revealing the molecular signature of neuropsychiatric disorders. *Braz. J. Psychiatry* 41 469–470. 10.1590/1516-4446-2019-0638 31826091 PMC6899348

[B227] Vogel Ciernia A. LaSalle J. (2016). The landscape of DNA methylation amid a perfect storm of autism aetiologies. *Nat. Rev. Neurosci.* 17 411–423. 10.1038/nrn.2016.41 27150399 PMC4966286

[B228] Volk L. Chiu S. L. Sharma K. Huganir R. L. (2015). Glutamate synapses in human cognitive disorders. *Annu. Rev. Neurosci.* 38 127–149. 10.1146/annurev-neuro-071714-033821 25897873

[B229] Wang A. M. Pradhan S. Coughlin J. M. Trivedi A. Dubois S. L. Crawford J. L. (2019). Assessing brain metabolism with 7-T proton magnetic resonance spectroscopy in patients with first-episode psychosis. *JAMA Psychiatry* 76 314–323. 10.1001/jamapsychiatry.2018.3637 30624573 PMC6439827

[B230] Wang M. Zhang L. Gage F. H. (2020). Modeling neuropsychiatric disorders using human induced pluripotent stem cells. *Protein Cell* 11 45–59. 10.1007/s13238-019-0638-8 31134525 PMC6949328

[B231] Wang W. Rein B. Zhang F. Tan T. Zhong P. Qin L. (2018). Chemogenetic activation of prefrontal cortex rescues synaptic and behavioral deficits in a mouse model of 16p11.2 deletion syndrome. *J. Neurosci.* 38 5939–5948. 10.1523/jneurosci.0149-18.2018 29853627 PMC6021990

[B232] Wei H. Zou H. Sheikh A. M. Malik M. Dobkin C. Brown W. T. (2011). IL-6 is increased in the cerebellum of autistic brain and alters neural cell adhesion, migration and synaptic formation. *J. Neuroinflamm.* 8:52. 10.1186/1742-2094-8-52 21595886 PMC3114764

[B233] Wei X. Ma T. Cheng Y. Huang C. C. Y. Wang X. Lu J. (2018). Dopamine D1 or D2 receptor-expressing neurons in the central nervous system. *Addict. Biol.* 23 569–584. 10.1111/adb.12512 28436559 PMC5654711

[B234] Whiteley J. T. Fernandes S. Sharma A. Mendes A. P. D. Racha V. Benassi S. K. (2022). Reaching into the toolbox: Stem cell models to study neuropsychiatric disorders. *Stem Cell Rep.* 17 187–210. 10.1016/j.stemcr.2021.12.015 35063127 PMC8828548

[B235] Williams S. E. Mealer R. G. Scolnick E. M. Smoller J. W. Cummings R. D. (2020). Aberrant glycosylation in schizophrenia: A review of 25 years of post-mortem brain studies. *Mol. Psychiatry* 25 3198–3207. 10.1038/s41380-020-0761-1 32404945 PMC8081047

[B236] Wright C. Turner J. A. Calhoun V. D. Perrone-Bizzozero N. (2013). Potential impact of miR-137 and Its targets in schizophrenia. *Front. Genet.* 4:58. 10.3389/fgene.2013.00058 23637704 PMC3636510

[B237] Wroolie T. E. Kenna H. A. Singh M. K. Rasgon N. L. (2015). Association between insulin resistance and cognition in patients with depressive disorders: Exploratory analyses into age-specific effects. *J. Psychiatr. Res.* 60 65–72. 10.1016/j.jpsychires.2014.10.001 25455511

[B238] Wysoczański T. Sokoła-Wysoczańska E. Pękala J. Lochyński S. Czyż K. Bodkowski R. (2016). Omega-3 fatty acids and their role in central nervous system - a review. *Curr. Med. Chem.* 23 816–831. 10.2174/0929867323666160122114439 26795198

[B239] Xu M. Y. Wong A. H. C. (2018). GABAergic inhibitory neurons as therapeutic targets for cognitive impairment in schizophrenia. *Acta Pharmacol. Sin.* 39 733–753. 10.1038/aps.2017.172 29565038 PMC5943898

[B240] Xu Z. Jiang H. Zhong P. Yan Z. Chen S. Feng J. (2016). Direct conversion of human fibroblasts to induced serotonergic neurons. *Mol. Psychiatry* 21 62–70. 10.1038/mp.2015.101 26216300 PMC4518549

[B241] Yamazaki M. Yamamoto N. Yarimizu J. Okabe M. Moriyama A. Furutani M. (2018). Functional mechanism of ASP5736, a selective serotonin 5-HT5A receptor antagonist with potential utility for the treatment of cognitive dysfunction in schizophrenia. *Eur. Neuropsychopharmacol.* 28 620–629. 10.1016/j.euroneuro.2018.03.003 29571967

[B242] Yang Y. Liu Y. Wang G. Hei G. Wang X. Li R. (2019). Brain-derived neurotrophic factor is associated with cognitive impairments in first-episode and chronic schizophrenia. *Psychiatry Res.* 273 528–536. 10.1016/j.psychres.2019.01.051 30710808

[B243] Yao B. Christian K. M. He C. Jin P. Ming G. L. Song H. (2016). Epigenetic mechanisms in neurogenesis. *Nat. Rev. Neurosci.* 17 537–549. 10.1038/nrn.2016.70 27334043 PMC5610421

[B244] Yap C. X. Henders A. K. Alvares G. A. Wood D. L. A. Krause L. Tyson G. W. (2021). Autism-related dietary preferences mediate autism-gut microbiome associations. *Cell* 184 5916–5931.e17. 10.1016/j.cell.2021.10.015 34767757

[B245] You M. J. Bang M. Park H. S. Yang B. Jang K. B. Yoo J. (2020). Human umbilical cord-derived mesenchymal stem cells alleviate schizophrenia-relevant behaviors in amphetamine-sensitized mice by inhibiting neuroinflammation. *Transl. Psychiatry* 10:123. 10.1038/s41398-020-0802-1 32341334 PMC7186225

[B246] Zafar S. Jabeen I. (2018). Structure, function, and modulation of γ-aminobutyric acid transporter 1 (GAT1) in neurological disorders: A pharmacoinformatic prospective. *Front. Chem.* 6:397. 10.3389/fchem.2018.00397 30255012 PMC6141625

[B247] Zalcman S. Green-Johnson J. M. Murray L. Nance D. M. Dyck D. Anisman H. (1994). Cytokine-specific central monoamine alterations induced by interleukin-1, -2 and -6. *Brain Res.* 643 40–49. 10.1016/0006-8993(94)90006-x 7518332

[B248] Zazula R. Dodd S. Dean O. M. Berk M. Bortolasci C. C. Verri W. A. Jr. (2022). Cognition-immune interactions between executive function and working memory, tumour necrosis factor-alpha (TNF-alpha) and soluble TNF receptors (sTNFR1 and sTNFR2) in bipolar disorder. *World J. Biol. Psychiatry* 23 67–77. 10.1080/15622975.2021.1925152 33949291

[B249] Zhang L. Zheng H. Wu R. Kosten T. R. Zhang X.-Y. Zhao J. (2019). The effect of minocycline on amelioration of cognitive deficits and pro-inflammatory cytokines levels in patients with schizophrenia. *Schizophr. Res.* 212 92–98. 10.1016/j.schres.2019.08.005 31416745

[B250] Zhang X. Yang M. Du X. Liao W. Chen D. Fan F. (2020). Glucose disturbances, cognitive deficits and white matter abnormalities in first-episode drug-naive schizophrenia. *Mol. Psychiatry* 25 3220–3230. 10.1038/s41380-019-0478-1 31409883

[B251] Zhang Y. Zhao Y. Song X. Luo H. Sun J. Han C. (2020). Modulation of stem cells as therapeutics for severe mental disorders and cognitive impairments. *Front. Psychiatry* 11:80. 10.3389/fpsyt.2020.00080 32425815 PMC7205035

[B252] Zheng P. Zeng B. Liu M. Chen J. Pan J. Han Y. (2019). The gut microbiome from patients with schizophrenia modulates the glutamate-glutamine-GABA cycle and schizophrenia-relevant behaviors in mice. *Sci. Adv.* 5:eaau8317. 10.1126/sciadv.aau8317 30775438 PMC6365110

[B253] Zhu F. Ju Y. Wang W. Wang Q. Guo R. Ma Q. (2020). Metagenome-wide association of gut microbiome features for schizophrenia. *Nat. Commun.* 11:1612. 10.1038/s41467-020-15457-9 32235826 PMC7109134

[B254] Zhu J. Hu W. Zhou Y. Qiao J. Chang X. Tong Z. (2019). Serum high-sensitivity C-reactive protein levels are positively associated with cognitive impairments in patients with first-episode schizophrenia. *Compr. Psychiatry* 94:152118. 10.1016/j.comppsych.2019.152118 31450022

