# Supplementary material for: Cognitive impairment in psychiatric diseases: Biomarkers of diagnosis, treatment, and prevention
Source: Front Cell Neurosci. 2022 Nov 2;16:1046692. doi: 10.3389/fncel.2022.1046692 (PMC9666407; doi:10.3389/fncel.2022.1046692)
Supplement: Supplementary file 1 [file Table_1.DOCX]

Supplementary Material

## Supplementary Table

| System | Mechanism/ Medication | Description | Disease | Targets for cognitive improvement | Reference |
| --- | --- | --- | --- | --- | --- |
| Glutamatergic system | 24(S)-hydroxycholesterol (24(S)-HC) | At submicromolar concentrations 24(S)-HC potentiates NMDAR-mediated EPSCs in rat hippocampal neurons but fails to affect AMPAR or GABAA receptors (GABA(A)Rs)-mediated responses. | SCZ;  ASD | NMDAR | (Paul et al., 2013, Burnell et al., 2019) |
|  | Memantine | The results of behavioral tests and cognitive function after memantine treatment were significantly normalized, and the copper concentration was decreased | MDD | NMDAR | (Li et al., 2020) |
| Dopaminergic system | dihydrexidine | Low doses can improve the work memory of monkeys. | SCZ | D1R | (Arnsten et al., 2017) |
|  | ASP4345 | A novel dopamine D1 receptor PAM is being evaluated for the treatment of cognitive impairment associated with schizophrenia | SCZ | D1R | (Desai et al., 2021) |
|  | GSK3β signaling | A glycogen synthase kinase-3 type enzyme that functions in ENERGY METABOLISM; EMBRYONIC DEVELOPMENT; and NEUROGENESIS. | BD | D3R | (Chang et al., 2020) |
|  | F17464 | 6-week trial demonstrated therapeutic efficacy of 40 mg/day F17464 in improving symptoms of acute exacerbation of schizophrenia with a favorable safety profile. | SCZ | D3R | (Bitter et al., 2019) |
|  | Stress exposure | Stress exposure in dopamine D4 receptor knockout mice induced significant deficits in schizophrenia-like behaviors | SCZ | D4R;GABA | (Tan et al., 2019) |
| GABA system | parvalbumin (PV) | Recent studies have revealed new facets of the development of PV neurons in primate neocortex and of the nature of their molecular alterations | SCZ | GABA | (Lewis, 2014, Lewis et al., 2012, Kim et al., 2016) |
|  | GABA inhibitory circuits | Deficits in GABA inhibitory circuits lead to impaired neural oscillations in schizophrenia | SCZ | GABA | (Lewis et al., 2008, Chen et al., 2014, Uhlhaas and Singer, 2010) |
|  | GABA transporter type 1 (GAT-1) | Alterations of GAT-1 genes related to GABA neurotransmission | SCZ | GABA | (Scheggia et al., 2018, Xu and Wong, 2018) |
|  | Excitatory/inhibitory (E/I) balance | Alterations in the balance between neuronal E/I balance have been implicated in the neural circuit activity-based processes that contribute to autism phenotypes. | ASD | GABA | (Selimbeyoglu et al., 2017) |
|  | Decreased PV, decreased mRNA encoding receptors for GABA, and decreased GABA/glutamate | These have been observed in autism patients and in mouse models. | ASD | GABA | (Hashemi et al., 2017, Fatemi et al., 2010, Alabdali et al., 2014, Chao et al., 2010) |
|  | Reelin and GAD 67 | Changes in GABAergic neuropathology, such as Reelin and GAD 67, have been detected in the hippocampus and cortex in the brains of patients with BD. | BD | GABA | (Fatemi et al., 2000, Guidotti et al., 2000, Veldic et al., 2007) |
|  | Decreased GABA Levels | Decreased GABA levels have been found in the prefrontal cortex, anterior cingulate cortex, cerebrospinal fluid, and plasma. | MDD | GABA | (Levinson et al., 2010, Northoff and Sibille, 2014, Fee et al., 2017, Klempan et al., 2009) |
| Serotonin system | 5-HT signaling | Increased 5-HT signaling is thought to be associated with schizophrenia and ASD, while decreased 5-HT signaling is thought to be associated with major depression and bipolar disorder. | MDD,  BD,  SCZ,  ASD | 5-HT | (Sumiyoshi et al., 1996, Selvaraj et al., 2014, Chen et al., 2017, Lemonde et al., 2003, Albert and Lemonde, 2004) |
|  | MIN-101 | A molecule that combines sigma-2 antagonism and 5-HT2A antagonism | SCZ | 5-HT2A receptor | (Keefe et al., 2018) |
|  | 5-HT6R antagonists | These compounds increase the number of NCAM PSA-immunoreactive neurons in the dendate gyrus, inhibit mTOR and Fyn-tyrosine kinase and interact with DARPP-32. | SCZ | 5-HT6R | (de Bruin and Kruse, 2015) |
|  | 5-HT-2AR antagonists | Risperidone and aripiprazole have the strongest evidence in reducing ABC-I in youth with ASD. | ASD | 5-HT2A receptor | (Fung et al., 2016, Amodeo et al., 2014) |
|  | ASP5736 | A novel and selective 5-HT5A receptor antagonist, exerts a positive effect in animal models of cognitive impairment. | SCZ | 5-HT5A receptor | (Yamazaki et al., 2018) |
|  | SSRIs | SSRIs are novel types of antidepressants, which can inhibit the reuptake of serotonin at synapses, thereby increasing the content of serotonin | MDD | serotonin | (Liu et al., 2021, Frampton, 2016) |

**Supplementary Table 1.** Neurotransmitter systems associated with cognitive impairment in psychiatric disorders.

Alabdali, A., Al-Ayadhi, L. & El-Ansary, A. (2014). Association of social and cognitive impairment and biomarkers in autism spectrum disorders. *J Neuroinflammation,* 11**,** 4.<https://doi.org/10.1186/1742-2094-11-4>

Albert, P. R. & Lemonde, S. (2004). 5-HT1A receptors, gene repression, and depression: guilt by association. *Neuroscientist,* 10**,** 575-93.<https://doi.org/10.1177/1073858404267382>

Amodeo, D. A., Jones, J. H., Sweeney, J. A. & Ragozzino, M. E. (2014). Risperidone and the 5-HT2A receptor antagonist M100907 improve probabilistic reversal learning in BTBR T + tf/J mice. *Autism Res,* 7**,** 555-67.<https://doi.org/10.1002/aur.1395>

Arnsten, A. F., Girgis, R. R., Gray, D. L. & Mailman, R. B. (2017). Novel Dopamine Therapeutics for Cognitive Deficits in Schizophrenia. *Biol Psychiatry,* 81**,** 67-77.<https://doi.org/10.1016/j.biopsych.2015.12.028>

Bitter, I., Lieberman, J. A., Gaudoux, F., Sokoloff, P., Groc, M., Chavda, R., et al. (2019). Randomized, double-blind, placebo-controlled study of F17464, a preferential D(3) antagonist, in the treatment of acute exacerbation of schizophrenia. *Neuropsychopharmacology,* 44**,** 1917-1924.<https://doi.org/10.1038/s41386-019-0355-2>

Burnell, E. S., Irvine, M., Fang, G., Sapkota, K., Jane, D. E. & Monaghan, D. T. (2019). Positive and Negative Allosteric Modulators of N-Methyl-d-aspartate (NMDA) Receptors: Structure-Activity Relationships and Mechanisms of Action. *J Med Chem,* 62**,** 3-23.<https://doi.org/10.1021/acs.jmedchem.7b01640>

Chang, P.-K., Chu, J., Tsai, Y.-T., Lai, Y.-H. & Chen, J.-C. (2020). Dopamine D3 receptor and GSK3β signaling mediate deficits in novel object recognition memory within dopamine transporter knockdown mice. *Journal of Biomedical Science,* 27**,** 16.<https://doi.org/10.1186/s12929-019-0613-y>

Chao, H. T., Chen, H., Samaco, R. C., Xue, M., Chahrour, M., Yoo, J., et al. (2010). Dysfunction in GABA signalling mediates autism-like stereotypies and Rett syndrome phenotypes. *Nature,* 468**,** 263-9.<https://doi.org/10.1038/nature09582>

Chen, C. M., Stanford, A. D., Mao, X., Abi-Dargham, A., Shungu, D. C., Lisanby, S. H., et al. (2014). GABA level, gamma oscillation, and working memory performance in schizophrenia. *Neuroimage Clin,* 4**,** 531-9.<https://doi.org/10.1016/j.nicl.2014.03.007>

Chen, R., Davis, L. K., Guter, S., Wei, Q., Jacob, S., Potter, M. H., et al. (2017). Leveraging blood serotonin as an endophenotype to identify de novo and rare variants involved in autism. *Mol Autism,* 8**,** 14.<https://doi.org/10.1186/s13229-017-0130-3>

De Bruin, N. M. & Kruse, C. G. (2015). 5-HT6 Receptor Antagonists: Potential Efficacy for the Treatment of Cognitive Impairment in Schizophrenia. *Curr Pharm Des,* 21**,** 3739-59.<https://doi.org/10.2174/1381612821666150605112105>

Desai, A., Benner, L., Wu, R., Gertsik, L., Maruff, P., Light, G. A., et al. (2021). Phase 1 randomized study on the safety, tolerability, and pharmacodynamic cognitive and electrophysiological effects of a dopamine D(1) receptor positive allosteric modulator in patients with schizophrenia. *Neuropsychopharmacology,* 46**,** 1145-1151.<https://doi.org/10.1038/s41386-020-00908-0>

Fatemi, S. H., Earle, J. A. & Mcmenomy, T. (2000). Reduction in Reelin immunoreactivity in hippocampus of subjects with schizophrenia, bipolar disorder and major depression. *Mol Psychiatry,* 5**,** 654-63, 571.<https://doi.org/10.1038/sj.mp.4000783>

Fatemi, S. H., Reutiman, T. J., Folsom, T. D., Rooney, R. J., Patel, D. H. & Thuras, P. D. (2010). mRNA and protein levels for GABAAalpha4, alpha5, beta1 and GABABR1 receptors are altered in brains from subjects with autism. *J Autism Dev Disord,* 40**,** 743-50.<https://doi.org/10.1007/s10803-009-0924-z>

Fee, C., Banasr, M. & Sibille, E. (2017). Somatostatin-Positive Gamma-Aminobutyric Acid Interneuron Deficits in Depression: Cortical Microcircuit and Therapeutic Perspectives. *Biol Psychiatry,* 82**,** 549-559.<https://doi.org/10.1016/j.biopsych.2017.05.024>

Frampton, J. E. (2016). Vortioxetine: A Review in Cognitive Dysfunction in Depression. *Drugs,* 76**,** 1675-1682.<https://doi.org/10.1007/s40265-016-0655-3>

Fung, L. K., Mahajan, R., Nozzolillo, A., Bernal, P., Krasner, A., Jo, B., et al. (2016). Pharmacologic Treatment of Severe Irritability and Problem Behaviors in Autism: A Systematic Review and Meta-analysis. *Pediatrics,* 137 Suppl 2**,** S124-35.<https://doi.org/10.1542/peds.2015-2851K>

Guidotti, A., Auta, J., Davis, J. M., Di-Giorgi-Gerevini, V., Dwivedi, Y., Grayson, D. R., et al. (2000). Decrease in reelin and glutamic acid decarboxylase67 (GAD67) expression in schizophrenia and bipolar disorder: a postmortem brain study. *Arch Gen Psychiatry,* 57**,** 1061-9.<https://doi.org/10.1001/archpsyc.57.11.1061>

Hashemi, E., Ariza, J., Rogers, H., Noctor, S. C. & Martínez-Cerdeño, V. (2017). The Number of Parvalbumin-Expressing Interneurons Is Decreased in the Prefrontal Cortex in Autism. *Cereb Cortex,* 27**,** 1931-1943.<https://doi.org/10.1093/cercor/bhw021>

Keefe, R. S. E., Harvey, P. D., Khan, A., Saoud, J. B., Staner, C., Davidson, M., et al. (2018). Cognitive Effects of MIN-101 in Patients With Schizophrenia and Negative Symptoms: Results From a Randomized Controlled Trial. *J Clin Psychiatry,* 79.<https://doi.org/10.4088/JCP.17m11753>

Kim, H., Ährlund-Richter, S., Wang, X., Deisseroth, K. & Carlén, M. (2016). Prefrontal Parvalbumin Neurons in Control of Attention. *Cell,* 164**,** 208-218.<https://doi.org/10.1016/j.cell.2015.11.038>

Klempan, T. A., Sequeira, A., Canetti, L., Lalovic, A., Ernst, C., Ffrench-Mullen, J., et al. (2009). Altered expression of genes involved in ATP biosynthesis and GABAergic neurotransmission in the ventral prefrontal cortex of suicides with and without major depression. *Mol Psychiatry,* 14**,** 175-89.<https://doi.org/10.1038/sj.mp.4002110>

Lemonde, S., Turecki, G., Bakish, D., Du, L., Hrdina, P. D., Bown, C. D., et al. (2003). Impaired repression at a 5-hydroxytryptamine 1A receptor gene polymorphism associated with major depression and suicide. *J Neurosci,* 23**,** 8788-99.<https://doi.org/10.1523/jneurosci.23-25-08788.2003>

Levinson, A. J., Fitzgerald, P. B., Favalli, G., Blumberger, D. M., Daigle, M. & Daskalakis, Z. J. (2010). Evidence of cortical inhibitory deficits in major depressive disorder. *Biol Psychiatry,* 67**,** 458-64.<https://doi.org/10.1016/j.biopsych.2009.09.025>

Lewis, D. A. (2014). Inhibitory neurons in human cortical circuits: substrate for cognitive dysfunction in schizophrenia. *Curr Opin Neurobiol,* 26**,** 22-6.<https://doi.org/10.1016/j.conb.2013.11.003>

Lewis, D. A., Cho, R. Y., Carter, C. S., Eklund, K., Forster, S., Kelly, M. A., et al. (2008). Subunit-selective modulation of GABA type A receptor neurotransmission and cognition in schizophrenia. *Am J Psychiatry,* 165**,** 1585-93.<https://doi.org/10.1176/appi.ajp.2008.08030395>

Lewis, D. A., Curley, A. A., Glausier, J. R. & Volk, D. W. (2012). Cortical parvalbumin interneurons and cognitive dysfunction in schizophrenia. *Trends Neurosci,* 35**,** 57-67.<https://doi.org/10.1016/j.tins.2011.10.004>

Li, Z., Wang, G., Zhong, S., Liao, X., Lai, S., Shan, Y., et al. (2020). Alleviation of cognitive deficits and high copper levels by an NMDA receptor antagonist in a rat depression model. *Compr Psychiatry,* 102**,** 152200.<https://doi.org/10.1016/j.comppsych.2020.152200>

Liu, L., Lv, X., Zhou, S., Liu, Q., Wang, J., Tian, H., et al. (2021). The effect of selective serotonin reuptake inhibitors on cognitive impairment in patients with depression: A prospective, multicenter, observational study. *J Psychiatr Res,* 141**,** 26-33.<https://doi.org/10.1016/j.jpsychires.2021.06.020>

Northoff, G. & Sibille, E. (2014). Why are cortical GABA neurons relevant to internal focus in depression? A cross-level model linking cellular, biochemical and neural network findings. *Mol Psychiatry,* 19**,** 966-977.<https://doi.org/10.1038/mp.2014.68>

Paul, S. M., Doherty, J. J., Robichaud, A. J., Belfort, G. M., Chow, B. Y., Hammond, R. S., et al. (2013). The major brain cholesterol metabolite 24(S)-hydroxycholesterol is a potent allosteric modulator of N-methyl-D-aspartate receptors. *J Neurosci,* 33**,** 17290-300.<https://doi.org/10.1523/jneurosci.2619-13.2013>

Scheggia, D., Mastrogiacomo, R., Mereu, M., Sannino, S., Straub, R. E., Armando, M., et al. (2018). Publisher Correction: Variations in Dysbindin-1 are associated with cognitive response to antipsychotic drug treatment. *Nat Commun,* 9**,** 3560.<https://doi.org/10.1038/s41467-018-06062-y>

Selimbeyoglu, A., Kim, C. K., Inoue, M., Lee, S. Y., Hong, A. S. O., Kauvar, I., et al. (2017). Modulation of prefrontal cortex excitation/inhibition balance rescues social behavior in CNTNAP2-deficient mice. *Sci Transl Med,* 9.<https://doi.org/10.1126/scitranslmed.aah6733>

Selvaraj, S., Arnone, D., Cappai, A. & Howes, O. (2014). Alterations in the serotonin system in schizophrenia: a systematic review and meta-analysis of postmortem and molecular imaging studies. *Neurosci Biobehav Rev,* 45**,** 233-45.<https://doi.org/10.1016/j.neubiorev.2014.06.005>

Sumiyoshi, T., Stockmeier, C. A., Overholser, J. C., Dilley, G. E. & Meltzer, H. Y. (1996). Serotonin1A receptors are increased in postmortem prefrontal cortex in schizophrenia. *Brain Res,* 708**,** 209-14.<https://doi.org/10.1016/0006-8993(95)01361-x>

Tan, T., Wang, W., Williams, J., Ma, K., Cao, Q. & Yan, Z. (2019). Stress Exposure in Dopamine D4 Receptor Knockout Mice Induces Schizophrenia-Like Behaviors via Disruption of GABAergic Transmission. *Schizophrenia Bulletin,* 45**,** 1012-1023.<https://doi.org/10.1093/schbul/sby163>

Uhlhaas, P. J. & Singer, W. (2010). Abnormal neural oscillations and synchrony in schizophrenia. *Nat Rev Neurosci,* 11**,** 100-13.<https://doi.org/10.1038/nrn2774>

Veldic, M., Kadriu, B., Maloku, E., Agis-Balboa, R. C., Guidotti, A., Davis, J. M., et al. (2007). Epigenetic mechanisms expressed in basal ganglia GABAergic neurons differentiate schizophrenia from bipolar disorder. *Schizophr Res,* 91**,** 51-61.<https://doi.org/10.1016/j.schres.2006.11.029>

Xu, M. Y. & Wong, A. H. C. (2018). GABAergic inhibitory neurons as therapeutic targets for cognitive impairment in schizophrenia. *Acta Pharmacol Sin,* 39**,** 733-753.<https://doi.org/10.1038/aps.2017.172>

Yamazaki, M., Yamamoto, N., Yarimizu, J., Okabe, M., Moriyama, A., Furutani, M., et al. (2018). Functional mechanism of ASP5736, a selective serotonin 5-HT5A receptor antagonist with potential utility for the treatment of cognitive dysfunction in schizophrenia. *European Neuropsychopharmacology: The Journal of the European College of Neuropsychopharmacology,* 28**,** 620-629.<https://doi.org/10.1016/j.euroneuro.2018.03.003>
